# Supplementary material for: Apical anchorage and stabilization of subpellicular microtubules by apical polar ring ensures Plasmodium ookinete infection in mosquito
Source: Nat Commun. 2022 Dec 3;13:7465. doi: 10.1038/s41467-022-35270-w (PMC9719560; doi:10.1038/s41467-022-35270-w)
Supplement: Supplementary file 1 — Supplementary Information [file 41467_2022_35270_MOESM1_ESM.pdf]

Supplemental Information

Apical anchorage and stabilization of subpellicular microtubules by apical polar ring ensures *Plasmodium* ookinete infection in mosquito

Pengge Qian[1], Xu Wang[1], Cuirong Guan[2], Xin Fang[1], Mengya Cai[1], Chuan-qi Zhong[1], Yong Cui[1], Yanbin Li[1], Luming Yao[1], Huiting Cui[1,*], Kai Jiang[2,*], Jing Yuan[1,*]

1. Supplementary Figures 1-11 and figure legends

2. Supplementary Table 1 Primers and oligonucleotides used in this study

3. Supplementary Table 2 List of genetically modified parasite strains used in this study

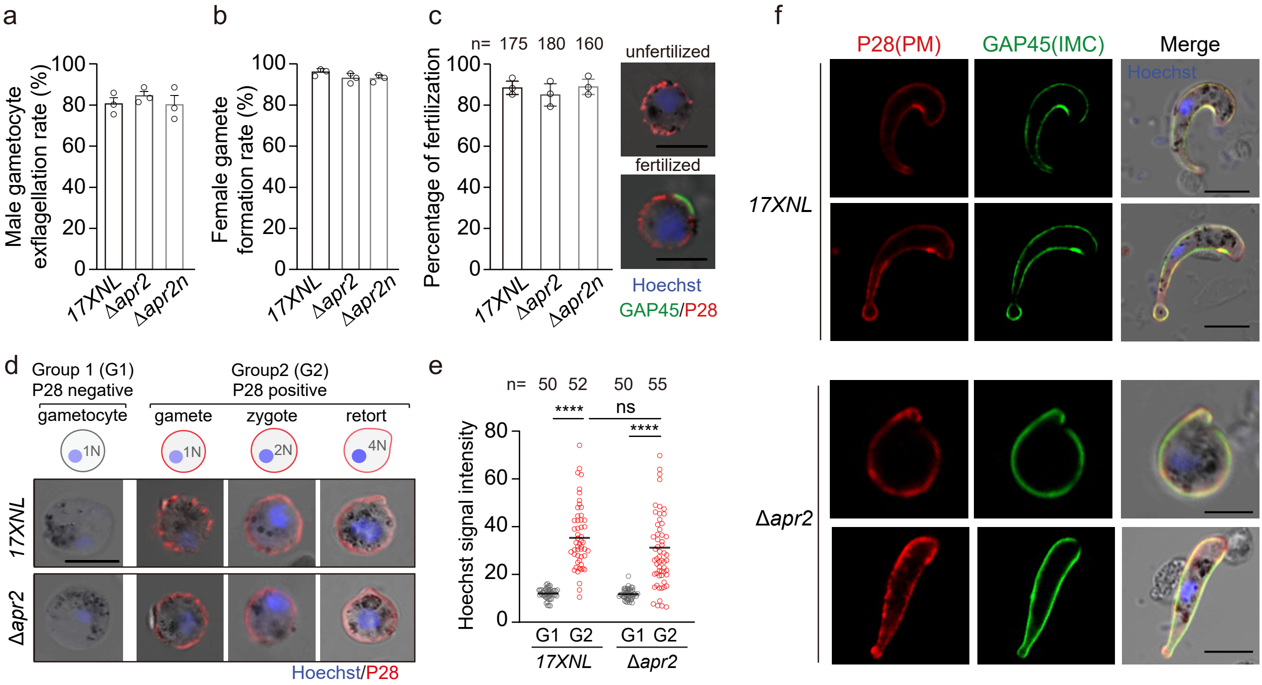
a
Male gametocyte exflagellation rate (%)
100
80
60
40
20
0
17XNL
Δapr2
Δapr2n
b
Female gamete formation rate (%)
17XNL
Δapr2
Δapr2n
c
n= 175 180 160
Percentage of fertilization
17XNL
Δapr2
Δapr2n
unfertilized
fertilized
Hoechst
GAP45/P28
d
Group 1 (G1)
P28 negative
Group2 (G2)
P28 positive
gametocyte
gamete
zygote
retort
1N
1N
2N
4N
17XNL
Δapr2
Hoechst/P28
e
n= 50 52 50 55
****
ns
****
Hoechst signal intensity
80
60
40
20
0
G1 G2 G1 G2
17XNL
Δapr2
f
P28(PM)
GAP45(IMC)
Merge
Hoechst
17XNL
Δapr2

Supplementary Figure 1. Phenotype analysis of the APR2-deficient parasites

a Male gamete formation *in vitro*. Male gamete formation rates are the percentage of male gametocytes showing exflagellation after xanthurenic acid (XA) stimulation. Values are means ± SD from three biological replicates.

b Female gamete formation *in vitro*. Female gamete formation rates are the percentage of female gametocytes showing P28 expression after XA stimulation. Values are means ± SD from three biological replicates.

c Gamete fertilization (zygote formation) *in vitro*. The parasites were co-stained with the antibodies against P28 (plasma membrane protein specific in female gamete, zygote, and ookinete) and GAP45 (IMC protein expressed in zygote and ookinete). Fertilization rate are the ratio of the numbers of cells displaying both $P28^{+}$ and $GAP45^{+}$ to the numbers of cells displaying only $P28^{+}$. n is the number of parasites analyzed. Means ± SD from three biological replicates. Representative images of female gamete and zygote from 17XNL parasites were shown in the right panels. Scale bars: 5 µm.

d Nuclei DNA content analysis of parasite post fertilization. A schematic in top panel indicates the genome DNA content change in the female gametocyte, female gamete, zygote, and retort/ookinete. One female gamete (1N) fertilizes with one male gamete to form zygote (2N) and further develops to retort/ookinete (4N) by meiotic DNA replication. The parasites were co-stained with anti-P28 antibody and DNA dye Hoechst 33342. Scale bars: 5 µm.

e Quantification of the Hoechst fluorescence signals in **d**. Data were pooled from three biological replicates, and values are means ± SD. n is the number of cells measured in each group. P value from left to right: ****, *P*=2e-20; ns, *P*=0.12; ****, *P*=7e-14; respectively, by One-way ANOVA followed by Tukey's multiple comparisons test.

f IFA of P28 (plasma membrane, PM) and GAP45 (inner membrane complex, IMC) of 17XNL and Δ*apr2* ookinetes. Scale bars: 5 µm. Two independently performed experiments with similar results.

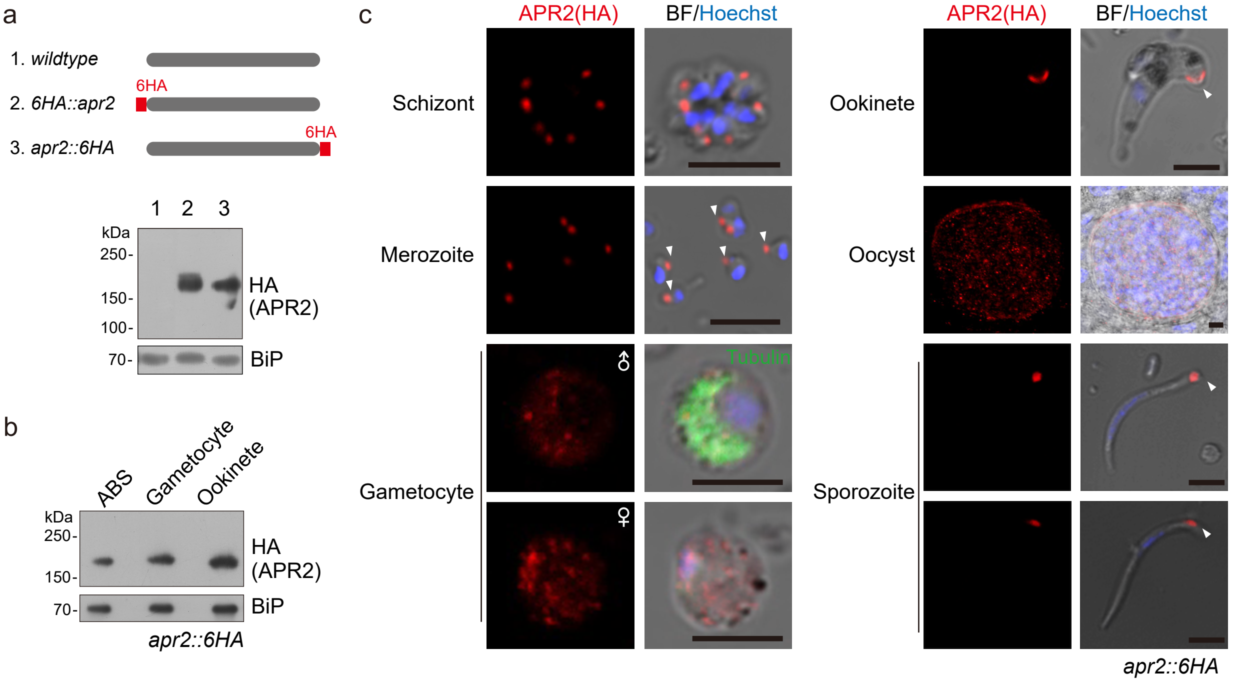
a
1. wildtype
6HA
2. 6HA::apr2
6HA
3. apr2::6HA
1 2 3
kDa
250-
150-
100-
70-
HA (APR2)
BiP
b
ABS
Gametocyte
Ookinete
kDa
250-
150-
70-
HA (APR2)
BiP
apr2::6HA
c
APR2(HA)
BF/Hoechst
Schizont
Merozoite
Gametocyte
♂
♀
Tubulin
APR2(HA)
BF/Hoechst
Ookinete
Oocyst
Sporozoite
apr2::6HA

Supplementary Figure 2. Stage expression and cellular localization of APR2

a Diagram showing 6HA-tagging of endogenous APR2 protein in the 17XNL parasite, generating two modified clones *6HA::apr2* and *apr2::6HA*. Immunoblot confirming the expression of APR2 in ookinetes of *6HA::apr2* and *apr2::6HA* parasites. Protein extract from 1.0×10^6 parasites was loaded for each sample. BiP serves as a loading control. Two independently performed experiments with similar results.

b Immunoblot of APR2 in the asexual blood stages (ABS), gametocytes, and ookinetes of the *apr2::6HA* parasite. Protein extract from 1.0×10^6 parasites was loaded for each sample. BiP serves as a loading control. Two independently performed experiments with similar results.

c IFA of APR2 at mouse and mosquito developmental stages of the *apr2::6HA* parasite. Gametocytes were co-stained with antibodies against HA (red) and α-Tubulin II (male gametocyte marker, green), while other parasite stages were stained with anti-HA antibody. White triangle indicated apical localization of APR2 in three zoites (merozoites, ookinetes, and sporozoites). Scale bars: 5 μm. Two independently performed experiments with similar results.

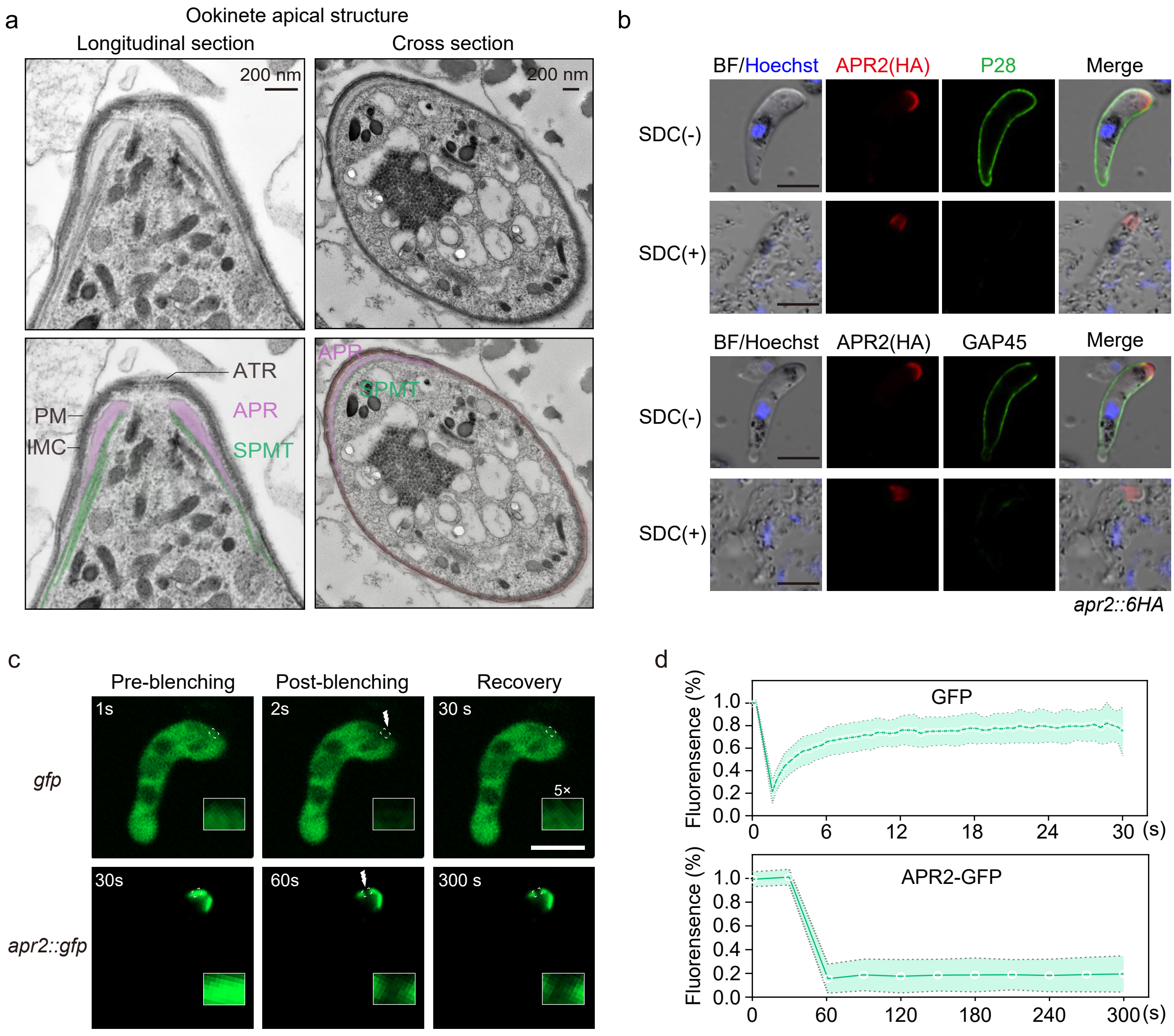

a
Ookinete apical structure
Longitudinal section
Cross section
200 nm
200 nm
ATR
APR
SPMT
PM
IMC
APR
SPMT
b
BF/Hoechst
APR2(HA)
P28
Merge
SDC(-)
SDC(+)
BF/Hoechst
APR2(HA)
GAP45
Merge
SDC(-)
SDC(+)
apr2::6HA
c
Pre-blenching
Post-blenching
Recovery
1s
2s
30 s
5×
gfp
30s
60s
300 s
apr2::gfp
d
GFP
Fluorensence (%)
1.0
0.8
0.6
0.4
0.2
0.0
0
6
12
18
24
30
(s)
APR2-GFP
Fluorensence (%)
1.0
0.8
0.6
0.4
0.2
0.0
0
60
120
180
240
300
(s)

Supplementary Figure 3. APR2 is associated with apical SPMTs in ookinetes

a Transmission electron microscopy (TEM) of apical longitudinal and cross sections of mature ookinete from 17XNL parasite. Original images were shown in the upper panel, while the modified images (with apical structures highlighted) were shown in the lower panel. ATR: apical tubulin ring; APR (magenta): apical polar ring; SPMT (green): subpellicular microtubule; PM: plasma membrane; IMC: inner membrane complex. Scale bars: 200 nm. Three independently performed experiments with similar results.

b IFA of P28 (plasma membrane protein) and GAP45 (inner membrane complex protein) in the ookinetes with or without the ionic detergent sodium deoxycholate (SDC) treatment. Scale bars: 5 μm. Three independently performed experiments with similar results.

c Fluorescence recovery after photobleaching (FRAP) of the GFP-tagged APR2 protein in living ookinetes of the *apr2::gfp* parasite. The parasite *gfp* is a control line with transgenic GFP expressing in the cytosol. The area for photobleaching was indicated by a box with white dash line and zoomed in. Scale bars: 5 μm. Three independently performed experiments with similar results.

d Quantification of fluorescence signal over time in **c** from three biological replicates. Data are shown in means ± SEM.

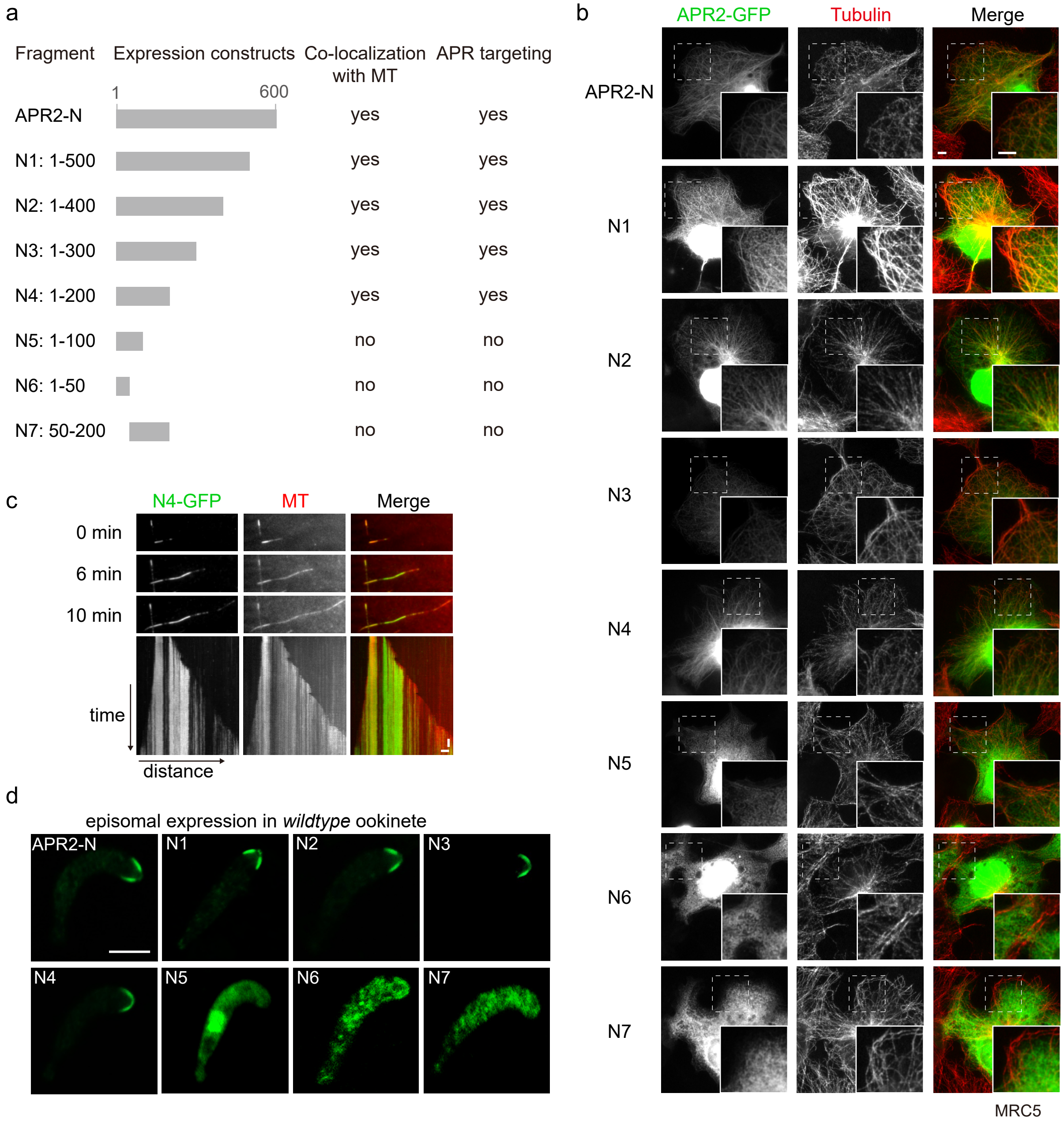
a
Fragment
Expression constructs
Co-localization with MT
APR targeting
1
600
APR2-N
yes
yes
N1: 1-500
yes
yes
N2: 1-400
yes
yes
N3: 1-300
yes
yes
N4: 1-200
yes
yes
N5: 1-100
no
no
N6: 1-50
no
no
N7: 50-200
no
no
b
APR2-GFP
Tubulin
Merge
APR2-N
N1
N2
N3
N4
N5
N6
N7
MRC5
c
N4-GFP
MT
Merge
0 min
6 min
10 min
time
distance
d
episomal expression in wildtype ookinete
APR2-N
N1
N2
N3
N4
N5
N6
N7

Supplementary Figure 4. APR2 N-terminal fragment (APR2-N) shows MT-binding and APR targeting

a Design and summary of APR2 N-terminal fragments for testing MT-binding and APR-targeting ability. APR2-N (1-600 aa) and another seven truncated fragments (N1-N7) were tested.

b IFA of APR2 fragments (GFP, green) and MTs (Tubulin, red) in the mammalian cell line MRC5. APR2-N and seven truncated fragments (N1-N7) were fused with GFP at the C-terminal and transiently expressed in the MRC5 cells. Scale bars: 5 μm. Three independently performed experiments with similar results.

c *In vitro* MT binding of proteins detected by total internal reflection fluorescence (TIRF) microscopy. Representative images and kymographs showed accumulation of APR2-N4 (green) on the growing MT (red) over time. Horizontal scale bars: 2 μm; vertical scale bars: 1 min.

d IFA of APR2 fragments episomally expressed in the ookinetes. APR2-N and seven truncated fragments (N1-N7) were fused with 6HA at the C-terminal and episomally expressed in *wildtype* ookinetes. Scale bars: 5 μm. Three independently performed experiments with similar results.

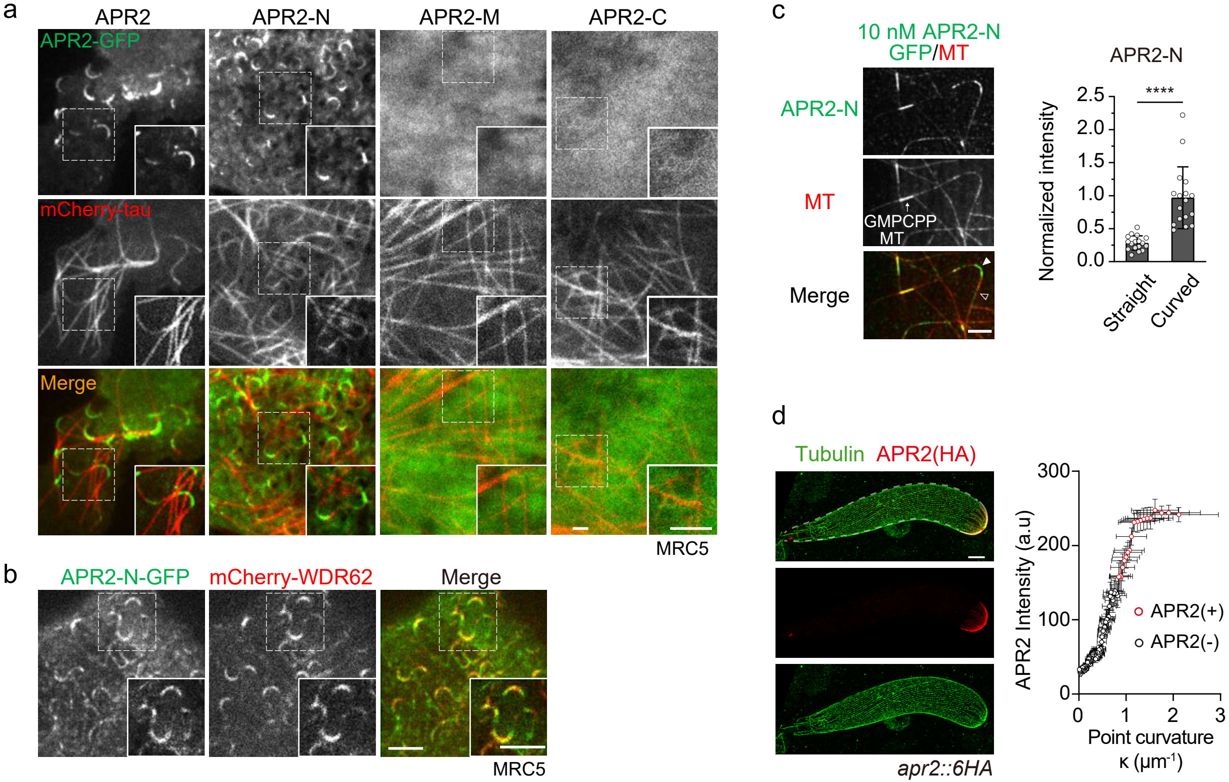

a
APR2
APR2-N
APR2-M
APR2-C
APR2-GFP
mCherry-tau
Merge
MRC5
b
APR2-N-GFP
mCherry-WDR62
Merge
MRC5
c
10 nM APR2-N
GFP/MT
APR2-N
MT
GMPCPP
MT
Merge
APR2-N
Normalized intensity
2.5
2.0
1.5
1.0
0.5
0.0
****
Straight
Curved
d
Tubulin
APR2(HA)
apr2::6HA
APR2 Intensity (a.u)
300
200
100
0
APR2(+)
APR2(-)
0
1
2
3
Point curvature
κ (μm-1)

Supplementary Figure 5. APR2-N prefers binding curved MT

a TIRF microscopy images of live MRC5 cells expressing APR2 fragments (GFP, green) and Tau (mCherry, red). Insets show enlargements of the boxed areas. APR2 and three fragments (APR2-N: 1-600 aa, APR2-M: 501-1100 aa, and APR2-C: 1001-1394 aa) were fused with GFP at the C-terminal and transiently expressed in the MRC5 cells with transgenic expression of mCherry-tagged Tau. Tau is a mammalian MT-binding protein for tracking MTs. Scale bars: 5 µm. Two independently performed experiments with similar results.

b TIRF microscopy images of live MRC5 cells expressing APR2-N (GFP, green) together with WDR62 (mCherry, red). Insets show enlargements of the boxed areas. WDR62 is a mammalian MT-binding protein and preferentially binds the curved MTs. Scale bars: 5 µm. Two independently performed experiments with similar results.

c TIRF microscopy images showing the preferential binding of GFP-tagged APR2-N (green) to curved MTs (red) in a flow-in assay. The arrow denotes the GMPCPP seed. Scale bars: 5 µm. Right panel indicated the quantification of the intensities of GFP-tagged APR2-N on straight (hollow triangle) and curved (solid triangle) segments of GDP lattices. The values were normalized to the intensity of curved segments. One representative experiments (n = 87 MTs) from three independent biological repeats was shown. ****, *P*=2e-06, two-sided *t* test.

d Plot of curvature distribution for SPMTs in the ookinetes. Values are means ± SD. The *apr2::6HA* ookinetes were imaged by U-ExM after staining with antibodies against HA (red) and Tubulin (α- and β-Tubulin, green), as shown in the left panel. The signal for APR2 (solid line) and SPMT (solid and dashed line) along cell periphery were tracked. Point curvature (κ) along the solid and dashed line was collected using the ImageJ plugin "Kappa". Representative experiment result (n = 20 ookinetes) from two independent biological repeats was shown. Scale bars: 1 µm. a.u: arbitrary units.

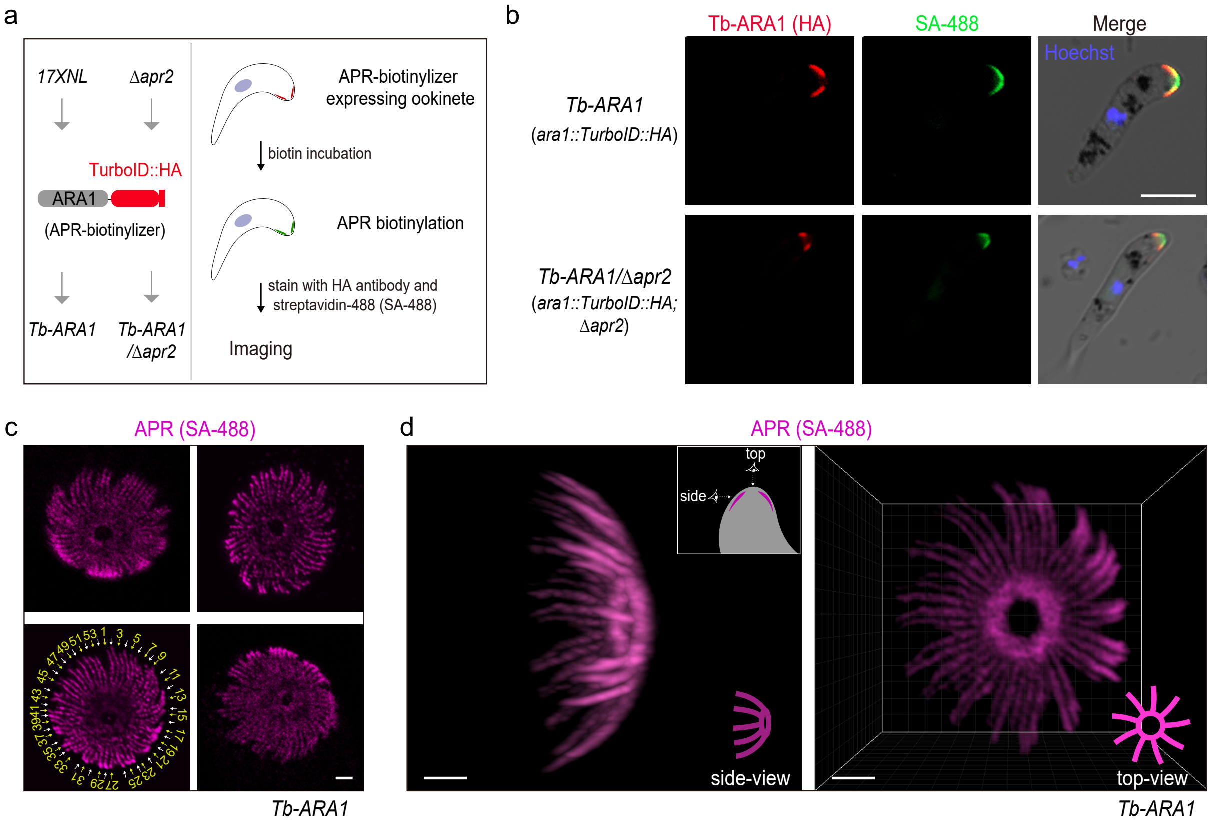
a
17XNL
Δapr2
TurboID::HA
ARA1
(APR-biotinylizer)
Tb-ARA1
Tb-ARA1
/Δapr2
APR-biotinylizer
expressing ookinete
biotin incubation
APR biotinylation
stain with HA antibody and
streptavidin-488 (SA-488)
Imaging
b
Tb-ARA1 (HA)
SA-488
Merge
Hoechst
Tb-ARA1
(ara1::TurboID::HA)
Tb-ARA1/Δapr2
(ara1::TurboID::HA;
Δapr2)
c
APR (SA-488)
Tb-ARA1
d
APR (SA-488)
top
side
side-view
top-view
Tb-ARA1

Supplementary Figure 6. Visualization of APR structure by TurboID-based proximity labeling and super-resolution imaging

a Flow-chart of proximity labeling and staining of APR in the APR-biotinylizer expressing ookinete. Endogenous ARA1 (APR-associated protein) was used as an APR-biotinylizer by fusing with a TurboID::HA motif in the 17XNL and Δ*apr2* parasites respectively, generating two modified lines *ara1::TurboID* (*Tb-ARA1*) and *ara1::TurboID*;Δ*apr2* (*Tb-ARA1/*Δ*apr2*). APR proteins undergo biotinylation by ARA1-fused TurboID in ookinetes incubated with biotin. APR biotinylation in the *Tb-ARA1* and *Tb-ARA1/*Δ*apr2* ookinetes was confirmed via IFA with streptavidin-488 (SA-488) and anti-HA antibody.

b IFA of HA-tagged ARA1 (red) and biotinylated proteins (green) in *Tb-ARA1* and *Tb-ARA1*/Δ*apr2* ookinetes. Note that a decrease in fluorescence signal area of biotinylizer and biotinylated proteins was observed after loss of APR2. Scale bars: 5 μm. Two independently performed experiments with similar results.

c Gallery of the PL-U-ExM images of APR (magenta) from the *Tb-ARA1* ookinetes after biotin-incubation and SA-488 staining. Maximum intensity projection (MIP) of SA-488 signal applied. The numbers (yellow) indicated the number of spines in APR. Scale bars: 0.5 μm. Three independently performed experiments with similar results.

d Representative PL-U-ExM images of APR (magenta) from side and top views of ookinete. Images were displayed using IMARIS in 3D blend mode. Scale bar: 0.5 μm. Three independently performed experiments with similar results.

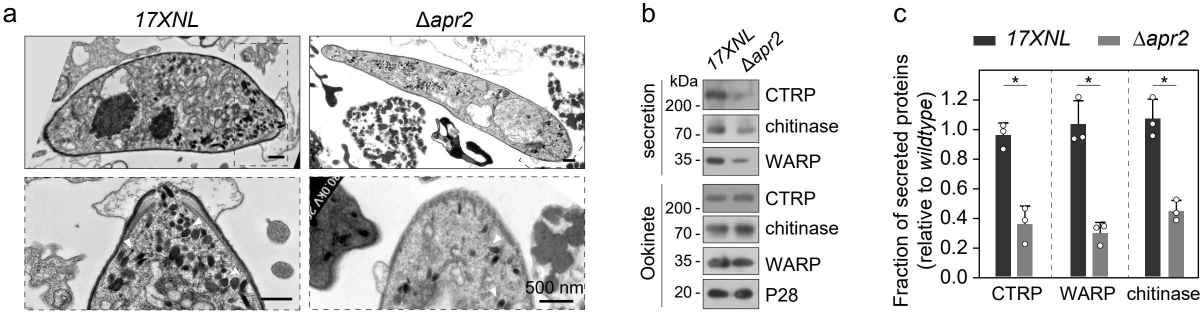
a
17XNL
Δapr2
500 nm
b
17XNL
Δapr2
kDa
secretion
Ookinete
200 -
70 -
35 -
200 -
70 -
35 -
20 -
CTRP
chitinase
WARP
CTRP
chitinase
WARP
P28
c
17XNL
Δapr2
Fraction of secreted proteins (relative to wildtype)
0.0
0.2
0.4
0.6
0.8
1.0
1.2
*
*
*
CTRP
WARP
chitinase

Supplementary Figure 7. Defective microneme protein secretion in the APR2-null ookinetes

a TEM longitudinal sections of 17XNL and Δ*apr2* ookinetes collected from 12 hours *in vitro* culture. The ookinete apical (dashed line in the upper panel) was zoomed in and shown in the lower panel. Scale bars: 0.5 μm. Two independently performed experiments with similar results.

b Immunoblot of three microneme-secreted proteins (CRTP, chitinase, and WARP) in ookinete culture supernatant and ookinete extract of 17XNL and Δ*apr*2 parasites. 3.0×10^6 ookinetes were lysed in each sample. Plasma membrane protein P28 as a loading control. Three independently performed experiments with similar results.

c Quantification of protein band intensity in **b**. Values are means ± SD from three biological replicates. P value from left to right: * P=0.04, * P=0.03, and * P=0.03, respectively, by two-sided paired t test.

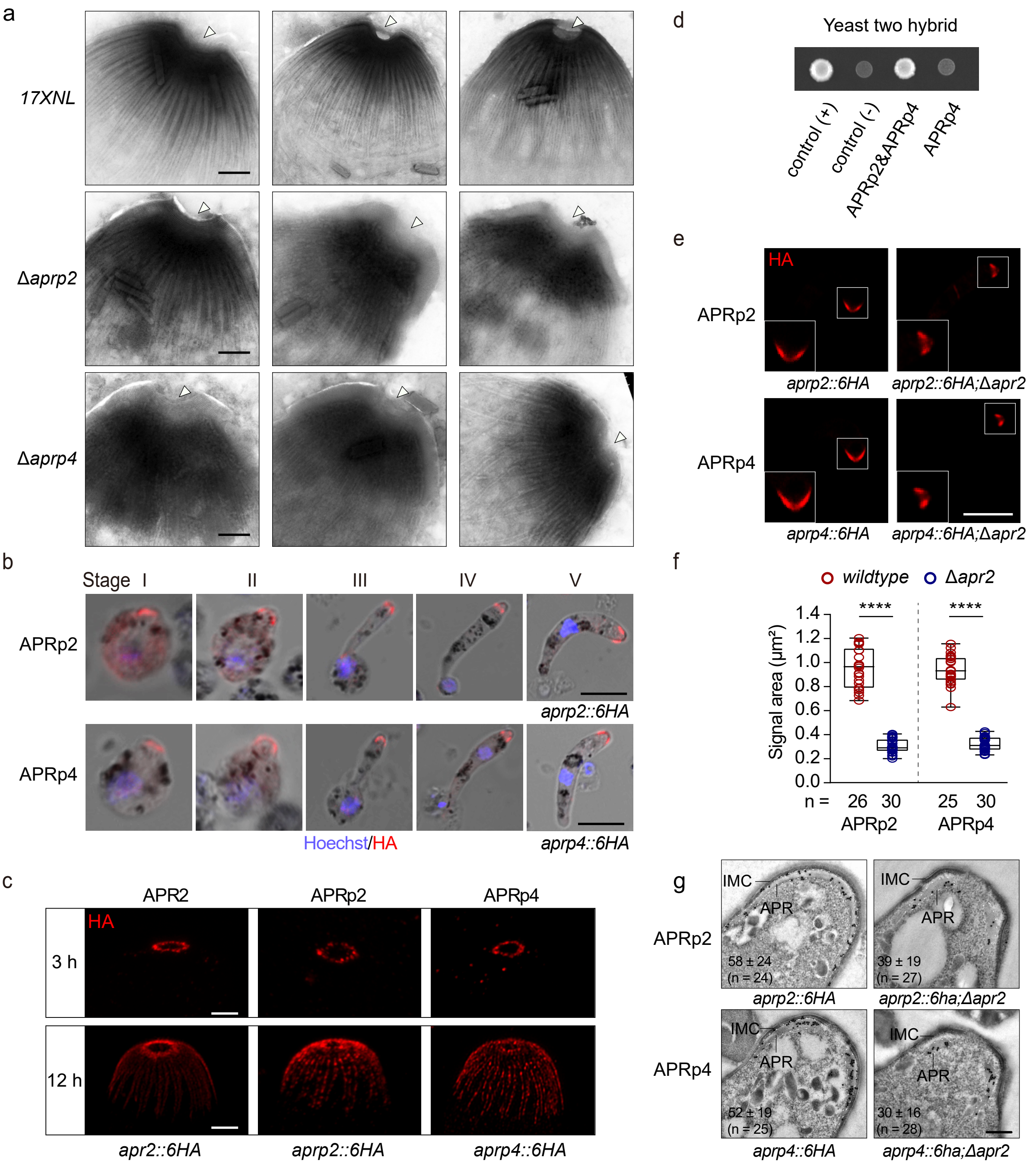
a
17XNL
Δaprp2
Δaprp4
b
Stage I
II
III
IV
V
APRp2
APRp4
aprp2::6HA
aprp4::6HA
Hoechst/HA
c
APR2
APRp2
APRp4
HA
3 h
12 h
apr2::6HA
aprp2::6HA
aprp4::6HA
d
Yeast two hybrid
control (+)
control (-)
APRp2&APRp4
APRp4
e
HA
APRp2
APRp4
aprp2::6HA
aprp2::6HA;Δapr2
aprp4::6HA
aprp4::6HA;Δapr2
f
wildtype
Δapr2
Signal area (μm²)
****
****
n = 26 30 25 30
APRp2
APRp4
g
IMC
APR
APRp2
APRp4
58 ± 24 (n = 24)
39 ± 19 (n = 27)
52 ± 19 (n = 25)
30 ± 16 (n = 28)
aprp2::6HA
aprp2::6ha;Δapr2
aprp4::6HA
aprp4::6ha;Δapr2

Supplementary Figure 8. APR2-APRp2-APRp4 module regulates apical anchorage of APR-SPMT

a Gallery of the NS-TEM images of 17XNL, Δ*aprp2* and Δ*aprp4* ookinete. A gap between SPMT and apical membrane-like layer was emerged in the Δ*aprp2* and Δ*aprp4* ookinetes, but not in 17XNL ookinetes. White triangles denote ookinete apical end. Scale bars: 0.5 µm. Three independently performed experiments with similar results.

b IFA of APRp2 and APRp4 expression through ookinete development of the HA-tagged strains *aprp2::6HA* and *aprp4::6HA*. Five stages (from I to V) were indicated. Scale bars: 5 µm. Two independently performed experiments with similar results.

c U-ExM analysis of HA-tagged APR2, APRp2 and APRp4 proteins in the *apr2::6HA*, *aprp2::6HA* and *aprp4::6HA* ookinetes respectively from 3- and 12-hour *in vitro* culture. Scale bars: 0.5 µm. Two independently performed experiments with similar results.

d Yeast two-hybrid (Y2H) assay detected interaction between APRp2 and APRp4. Interaction was detected in the group co-transfected with APRp2 and APRp4, but not in the group with only APRp4. Yeast expressing paired bait-pray constructs were grown under the restrictive conditions (SD: synthetic dropout medium). Representative results from two independent experiments. +, positive control; -, negative control.

e IFA of HA-tagged APRp2 and APRp4 in ookinetes in the presence and absence of APR2. Endogenous *apr2* gene was deleted by CRISPR-Cas9 in *aprp2::6HA* and *aprp4::6HA* parental parasites, generating *aprp2::6HA*;Δ*apr2* and *aprp4::6HA*;Δ*apr2* mutants. Insects represent enlarged views of the boxed areas. Scale bars: 5 µm. Representative results from two independent experiments.

f Quantification of IFA signal area in **e**. n is the number of cells tested in each group over two independent experiments. Boxes show medians with interquartile ranges, whiskers: min to max show all points. P value from left to right: ****, *P*=3e-27, and ****, *P*=4e-31, respectively, by two-sided Mann-Whitney test.

g Immuno-EM images of HA-tagged APRp2 and APRp4 in ookinetes in the presence and absence of APR2. Scale bars: 0.5 µm. The number (means ± SD) of colloidal gold particles per ookinete and the number (n) of ookinetes analyzed were shown.

Plasmodium ookinete morphogenesis for mosquito infection

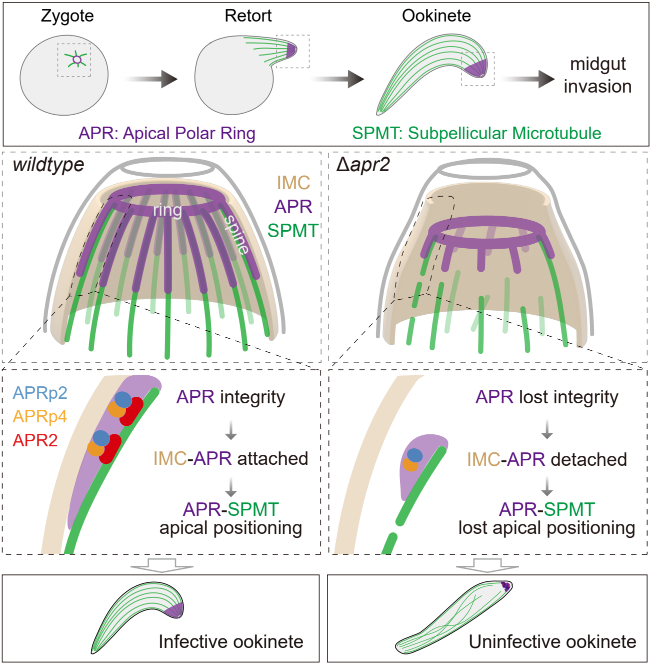

Supplementary Figure 9. A proposed model for APR2-APRp2-APRp4 module regulating the integrity and apical anchorage of APR-SPMT in ookinetes

a

	Rodent malaria parasites			Human malaria parasites		
	P.yoelii	***P.berghei***	***P.chabaudi***	***P.falciparum***	***P.vivax***	***P.malariae***
APR2	PY17X_1339500	PBANKA_1334800	PCHAS_1339400	PF3D7_1471600	PVP01_1235200	PmUG01_12045300
ARA1	PY17X_1412750	PBANKA_1410950	PCHAS_1412850	PF3D7_1312450	PVP01_1413450	PmUG01_14029200
APRp1	PY17X_0721000	PBANKA_0721000	PCHAS_0730000	PF3D7_0418900	PVP01_0528300	PmUG01_05036400
APRp2	PY17X_1322400	PBANKA_1318600	PCHAS_1321900	PF3D7_1454900	PVP01_1252100	PmUG01_12062100
APRp3	PY17X_0402200	PBANKA_1040300	PCHAS_1467400	-	-	-
APRp4	PY17X_1322300	PBANKA_1318500	PCHAS_1321800	PF3D7_1454800	PVP01_1252200	PmUG01_12062200
APRp5	PY17X_0807500	PBANKA_0804400	PCHAS_0804700	PF3D7_0707200	PVP01_0107400	PmUG01_01018700
APRp6	PY17X_1336300	PBANKA_1331600	PCHAS_1336200	PF3D7_1468300	PVP01_1238500	PmUG01_12048600
APRp7	PY17X_1111000	PBANKA_1109900	PCHAS_1109600	PF3D7_0510300	PVP01_1023700	PmUG01_06014600
APRp8	PY17X_0612100	PBANKA_0609600	PCHAS_0611300	PF3D7_1211100	PVP01_1310300	PmUG01_13021100
APRp9	PY17X_1322800	PBANKA_1319000	PCHAS_1322300	PF3D7_1455300	PVP01_1251600	PmUG01_12061600

b

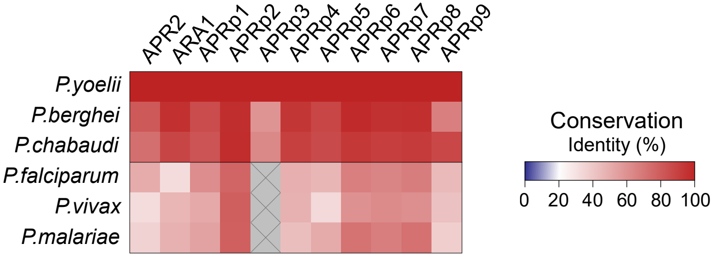

Supplementary Figure 10. Conservation of APR proteins in the rodent and human malaria parasites

a Gene orthologous of eleven APR proteins among six parasites including *P. yoelii*, *P. berghei*, *P. chabaudi*, *P. falciparum*, *P. vivax*, and *P. malariae*.

b Heatmap showing the conservation of APR proteins. Percentage of protein amino acid sequence identities were generated via the BlastP.

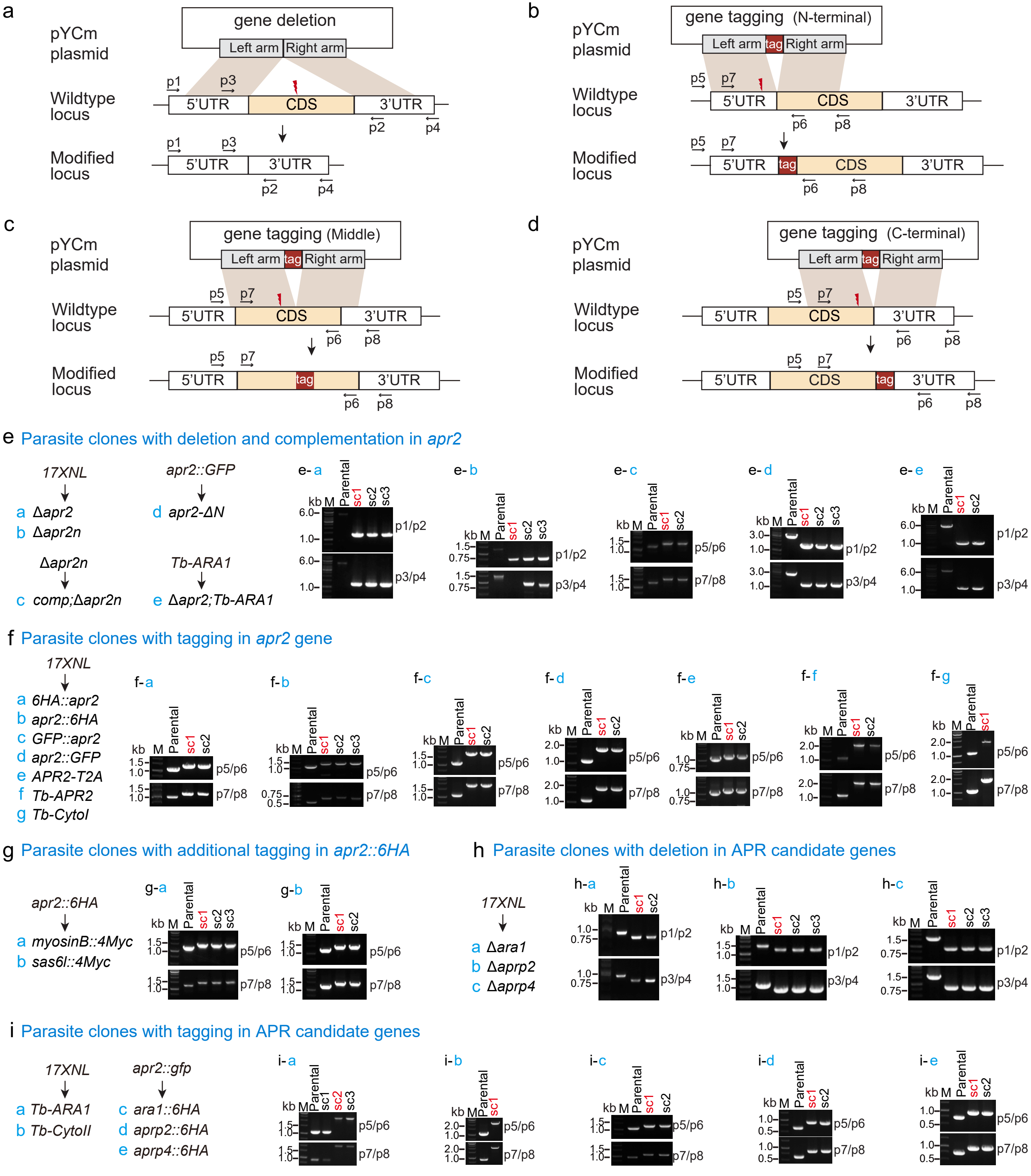

a
pYCm plasmid
gene deletion
Left arm
Right arm
Wildtype locus
5'UTR
CDS
3'UTR
Modified locus
p1 p2 p3 p4
b
gene tagging (N-terminal)
tag
p5 p6 p7 p8
c
gene tagging (Middle)
d
gene tagging (C-terminal)
e Parasite clones with deletion and complementation in apr2
17XNL
a Δapr2
b Δapr2n
Δapr2n
c comp;Δapr2n
apr2::GFP
d apr2-ΔN
Tb-ARA1
e Δapr2;Tb-ARA1
e-a e-b e-c e-d e-e
kb M Parental sc1 sc2 sc3
f Parasite clones with tagging in apr2 gene
17XNL
a 6HA::apr2
b apr2::6HA
c GFP::apr2
d apr2::GFP
e APR2-T2A
f Tb-APR2
g Tb-CytoI
f-a f-b f-c f-d f-e f-f f-g
g Parasite clones with additional tagging in apr2::6HA
apr2::6HA
a myosinB::4Myc
b sas6l::4Myc
g-a g-b
h Parasite clones with deletion in APR candidate genes
17XNL
a Δara1
b Δaprp2
c Δaprp4
h-a h-b h-c
i Parasite clones with tagging in APR candidate genes
17XNL
a Tb-ARA1
b Tb-CytoII
apr2::gfp
c ara1::6HA
d aprp2::6HA
e aprp4::6HA
i-a i-b i-c i-d i-e
p1/p2 p3/p4 p5/p6 p7/p8

Supplementary Figure 11. Genotyping of genetically modified parasites in this study

a-d Schematic representation for CRISPR-Cas9 mediated gene modification, including gene deletion (**a**), gene tagging at the N-terminus (**b**), gene tagging at the middle (**c**), and gene tagging at the C-terminus (**d**), via double cross homologous recombination.

e-i For each modification, both 5' and 3' homologous recombination were detected by the genotyping PCR (see the primers in the Supplementary Table 3) to confirm successful integration of the homologous templates. Usually, 1-3 parasite single clones (sc) were obtained after limiting dilution, and one clone (indicated with red letter) was used for further phenotype and gene functional analysis.

Supplementary Table 1. Primers and oligonucleotides used in this study

Primers for gene knockout									
Gene name	Gene ID	Modified strain	Gene size /deleted gene size (bp)	Left homologous template		Right homologous template		Target site of sgRNA	
				Forward primer	Reverse primer	Forward primer	Reverse primer	Oligo (Forward)	Oligo (Reverse)
apr2	PY17X_1339500	Δ*apr2*	4185/4185	CGGGGTACCGAGCTACAGTCCTATGAAATTGT	CATGCCATGGTCTCAtAGCGCCGAGACACT	CATGCCATGGGCAACCAAATAAGTAAAAAGAAT	CCCCTTAAGCCTGATTTTTACATATGTTGT	TATTCTAGTATATCCCTTCCCGGC	AAACGCCGGGAAGGGATATACTAG
apr2	PY17X_1339500	Δ*apr2n*	4285/601	CGGGGTACCGAGCTACAGTCCTATGAAATTGT	CATGCCATGGAGCGCCGAGACACTAGTAAA	CATGCCATGGTTAATAATGTCCAAATTAAAGCAGCT	CCCCTTAAGCTACAAGACTGTCAGAGGATACATTCTT	TATTTCTAAGAGGCGTTGATGGTT	AAACAACCATCAACGCCTCTTAGA
apr2	PY17X_1339500	*APR2-ΔN*	4285/1797	CGGGGTACCGAGCTACAGTCCTATGAAATTGT	CATGCCATGGCATAGCGCCGAGACACTAGTAAA	CATGCCATGGGATTTCCCCGTAAATAACAAAGT	CCCCTTAAGTTGGTTATTTTGTGGTATAGGAT	TATTTCTAAGAGGCGTTGATGGTT	AAACAACCATCAACGCCTCTTAGA
ara1	PY17X_1412750	Δ*ara1*	192/192	CGGGGTACCGCTTGGTATTATTCTGTCTA	CATGCCATGGTTTTGCAAATTTGGGAATTT	CCGCTCGAGTGTATTGAGTAATGAACCAC	CCCCTTAAGTGCTCATATTCATCCACGAT	TATTACGGCTGGTGTTGACACAAC	AAACGTTGTGTCAACACCAGCCGT
aprp2	PY17X_1322400	Δ*aprp2*	459/459	CGGGGTACCCTACAAATTAGTATGACGTGTACAT	CATGCCATGGGTTGCATCGTGAAAAATAGT	CCGCTCGAGGGATGTAATAGAATATCCCTAATCAT	CCCCTTAAGTACGACGATATATCGCCAAACT	TATTCAATGCAACAATTTCAAATG	AAACCATTTGAAATTGTTGCATTG
aprp4	PY17X_1214800	Δ*aprp4*	2529/2529	CCCAAGCTTGTGACGTGTACTTTCTTAACT	CATGCCATGGGCATAAATACTTATGACGTTGTGT	CCGCTCGAGCTTTCTGTGAAAGCAAACGAT	CCCCTTAAGAGTTTGGCGATATATCGTCGTATAT	TATTTGTCTAACAATTATTGGTCT	AAACAGACCAATAATTGTTAGACA
**Primers for PCR-genotyping parasite with gene knockout**									
**Gene name**	**Gene ID**	**Modified strain**	**P1**	**P2**	**P3**	**P4**			
apr2	PY17X_1339500	Δ*apr2*	GAATATCTTAACTTGTATAGCT	CCTGATTTTTACATATGTTGT	GAGCTACAGTCCTATGAAATTGT	TTATTGTCGTCTCCATTCAT			
apr2	PY17X_1339500	Δ*apr2n*	GAATATCTTAACTTGTATAGCT	CTACAAGACTGTCAGAGGAT	GAGCTACAGTCCTATGAAATTGT	TATGGGCTTAAATCGTTCAT			
apr2	PY17X_1339500	*APR2-ΔN*	GAATATCTTAACTTGTATAGCT	TTGGTTATTTTGTGGTATAGGAT	GAGCTACAGTCCTATGAAATTGT	TATTTGCACTGTTTGGGATT			
ara1	PY17X_1412750	Δ*ara1*	GCTAGATCTTTATTACACACACT	GCTCATATTCATCCACGATA	GCTTGGTATTATTCTGTCTA	GAGGCAAAGTATATCACATA			
aprp2	PY17X_1322400	Δ*aprp2*	TATATTTGTAAGGTGTGTAT	TACGACGATATATCGCCAAACT	CTACAAATTAGTATGACGTGTACAT	GCTATTTTCGTTTAATGTGTC			
aprp4	PY17X_1214800	Δ*aprp4*	GTTTGGTGAATAATAATTAGT	ACGTTTTCTTGGAATATATATT	GTGACGTGTACTTTCTTAACT	TACCTTGTGTAAATAAGATAT			
**Primers for gene complementation**									
**Gene name**	**Gene ID**	**Modified strain**	**Gene size /re-introduced gene size (bp)**	**Left homologous template**		**Right homologous template**		**Target site of sgRNA**	
				**Forward primer**	**Reverse primer**	**Forward primer**	**Reverse primer**	**Oligo (Forward)**	**Oligo (Reverse)**
apr2	PY17X_1339500	*comp*	4285/601	CGGGGTACCGAGCTACAGTCCTATGAAATtgt	CATGCCATGGAGCGCCGAGACACTAGTAAA	CATGCCATGGgaATGAGAAAAAAAAGTGAAATTT	CCCCTTAAGCTACAAGACTGTCAGAGGAT	TATTGCGGACGCTAATCGTAGCTA	AAACTAGCTACGATTAGCGTCCGC
**Primers for PCR-genotyping parasite with gene complementation**									
**Gene name**	**Gene ID**	**Modified strain**	**Gene size /re-introduced gene size (bp)**	**P1**	**P2**	**P3**	**P4**		
apr2	PY17X_1339500	*comp*	4285/601	GAATATCTTAACTTGTATAGCT	CTACAAGACTGTCAGAGGAT	GCTACAGTCCTATGAAATTGT	TATGGGCTTAAATCGTTCAT		
**Primers for gene tagging**									
**Gene name**	**Tag (name and location)**	**Modified strain**	**Gene ID**	**Left homologous template**		**Right homologous template**		**Target site of sgRNA**	
				**Forward primer**	**Rerverse primer**	**Forward primer**	**Reverse primer**	**Oligo (Forward)**	**Oligo (Reverse)**
apr2	C-terminal 6HA	*apr2::6HA*	PY17X_1339500	CGGGGTACCGGTACCATCAAATCGAATTAAT	CATGCCATGGGTTTAAATGACGTGATGCATAT	CCGCTCGAGGCAACCAAATAAGTAAAAAGAAT	CCCCTTAAGCCTGATTTTTACATATGTTGT	TATTCTAGTATATCCCTTCCCGGC	AAACGCCGGGAAGGGATATACTAG
apr2	N-terminal 6HA	*6HA::apr2*	PY17X_1339500	CGGGGTACCGAGCTACAGTCCTATGAAATTGT	CATGCCATGGAGCGCCGAGACACTAGTAAA	CCGCTCGAGATGAGAAAAAAAAGTGAAATTTCCAAA	CCCCTTAAGATGGAACAAATTGTTGGAAGCT	TATTTCTAAGAGGCGTTGATGGTT	AAACAACCATCAACGCCTCTTAGA
apr2	C-terminal GFP	*apr2::gfp*	PY17X_1339500	CGGGGTACCGGTACCATCAAATCGAATTAAT	CATGCCATGGGTTTAAATGACGTGATGCATAT	CCGCTCGAGGCAACCAAATAAGTAAAAAGAAT	CCCCTTAAGCCTGATTTTTACATATGTTGT	TATTCTAGTATATCCCTTCCCGGC	AAACGCCGGGAAGGGATATACTAG
apr2	N-terminal GFP	*gfp::apr2*	PY17X_1339500	CGGGGTACCGAGCTACAGTCCTATGAAATTGT	CATGCCATGGAGCGCCGAGACACTAGTAAA	CCGCTCGAGATGAGAAAAAAAAGTGAAATTTCCAAA	CCCCTTAAGATGGAACAAATTGTTGGAAGCT	TATTTCTAAGAGGCGTTGATGGTT	AAACAACCATCAACGCCTCTTAGA
apr2	T2A inserted between aa 845-846	*APR2-T2A*	PY17X_1339500	CGGGGTACCAATACCTATAATACCAATACAAG	CATGCCATGGTTGTGATACTGGAATTTGAGT	CATGCCATGGGGATTTACTCATGAAACACCAC	CCCCTTAAGCCGGTAGGTGAGCAGGTTTGT	TATTGTTGCATGAGTAAATCCTTG	AAACCAAGGATTTACTCATGCAAC
apr2	C-terminal Turboid-HA	*Tb-APR2*	PY17X_1339500	CGGGGTACCGGTACCATCAAATCGAATTAAT	CATGCCATGGGTTTAAATGACGTGATGCATAT	CCGCTCGAGGCAACCAAATAAGTAAAAAGAAT	CCCCTTAAGCCTGATTTTTACATATGTTGT	TATTCTAGTATATCCCTTCCCGGC	AAACGCCGGGAAGGGATATACTAG
apr2	C-terminal T2A-Turboid-HA	*Tb-cytoI*	PY17X_1339500	CGGGGTACCGGTACCATCAAATCGAATTAAT	CATGCCATGGGTTTAAATGACGTGATGCATAT	CCGCTCGAGGCAACCAAATAAGTAAAAAGAAT	CCCCTTAAGCCTGATTTTTACATATGTTGT	TATTCTAGTATATCCCTTCCCGGC	AAACGCCGGGAAGGGATATACTAG
ara1	C-terminal Turboid-HA	*Tb-ARA1*	PY17X_1412750	CGGGGTACCTGTAGGTAGCTATTTAGTT	CATGCCATGGTATTATTATGGTTTTTCCTCCTAT	CCGCTCGAGTGTATTGAGTAATGAACCAC	CCCCTTAAGTGCTCATATTCATCCACGATA	TATTACGGCTGGTGTTGACACAAC	AAACGTTGTGTCAACACCAGCCGT
ara1	C-terminal T2A-Turboid-HA	*Tb-cytoII*	PY17X_1412750	CGGGGTACCTGTAGGTAGCTATTTAGTT	CATGCCATGGTATTATTATGGTTTTTCCTCCTAT	CCGCTCGAGTGTATTGAGTAATGAACCAC	CCCCTTAAGTGCTCATATTCATCCACGATA	TATTACGGCTGGTGTTGACACAAC	AAACGTTGTGTCAACACCAGCCGT
ara1	C-terminal 6HA	*ara1::6HA*	PY17X_1412750	CGGGGTACCTGTAGGTAGCTATTTAGTT	CATGCCATGGTATTATTATGGTTTTTCCTCCTAT	CCGCTCGAGTGTATTGAGTAATGAACCAC	CCCCTTAAGTGCTCATATTCATCCACGATA	TATTACGGCTGGTGTTGACACAAC	AAACGTTGTGTCAACACCAGCCGT
aprp2	C-terminal 6HA	*aprp2::6HA*	PY17X_1322400	CGGGGTACCTTATTAAACTATTTTTCACGAT	CATGCCATGGCATTTGAATATTTTTATTCAAGT	CCGCTCGAGTCAAATATGGATGTAATAGAATAT	CCCCTTAAGTACGACGATATATCGCCAAA	TATTCAATGCAACAATTTCAAATG	AAACCATTTGAAATTGTTGCATTG
aprp4	C-terminal 6HA	*aprp4::6HA*	PY17X_1214800	CGGGGTACCATGGATAATTTATTATATTACAT	CATGCCATGGCCCTGTATGATTATTTGAATATT	CCGCTCGAGGATTAAGTACCTTAATTGCAT	CCCCTTAAGAGTTTGGCGATATATCGTCGTATAT	TATTTGTCTAACAATTATTGGTCT	AAACAGACCAATAATTGTTAGACA
**Primer sequence for PCR-genotyping parasite with gene tagging**									
**Gene name**	**Tag (name and location)**	**Modified strain**	**Gene ID**	**P1**	**P2**	**P3**	**P4**		
apr2	C-terminal 6HA	*apr2::6HA*	PY17X_1339500	GATATGGGGATGAACTTTGT	CCTGATTTTTACATATGTTGT	GGTACCATCAAATCGAATTAAT	TTATTGTCGTCTCCATTCAT		
apr2	N-terminal 6HA	*6HA::apr2*	PY17X_1339500	GAATATCTTAACTTGTATAGCT	ATGGAACAAATTGTTGGAAGCT	GAGCTACAGTCCTATGAAATTGT	ATGGACTTCAGGTTTTTCAGA		
apr2	C-terminal GFP	*apr2::gfp*	PY17X_1339500	GATATGGGGATGAACTTTGT	CCTGATTTTTACATATGTTGT	GGTACCATCAAATCGAATTAAT	TTATTGTCGTCTCCATTCAT		
apr2	N-terminal GFP	*gfp::apr2*	PY17X_1339500	GAATATCTTAACTTGTATAGCT	ATGGAACAAATTGTTGGAAGCT	GAGCTACAGTCCTATGAAATTGT	ATGGACTTCAGGTTTTTCAGA		
apr2	T2A inserted between 845-846 aa	*APR2-T2A*	PY17X_1339500	AATCCCAAACAGTGCAAATA	CCGGTAGGTGAGCAGGTTTGTTAT	AATACCTATAATACCAATACAAGCACAT	TATTTGGATATGCGCACACT		
apr2	C-terminal Turboid-HA	*Tb-APR2*	PY17X_1339500	GATATGGGGATGAACTTTGT	CCTGATTTTTACATATGTTGT	GGTACCATCAAATCGAATTAAT	TTATTGTCGTCTCCATTCAT		
apr2	C-terminal T2A-Turboid-HA	*Tb-cytoI*	PY17X_1339500	GATATGGGGATGAACTTTGT	CCTGATTTTTACATATGTTGT	GGTACCATCAAATCGAATTAAT	TTATTGTCGTCTCCATTCAT		
ara1	C-terminal Turboid-HA	*Tb-ARA1*	PY17X_1412750	GGAAAAAATGTATGCACAAC	TGCTCATATTCATCCACGATA	TGTAGGTAGCTATTTAGTT	ATCCATTTGAAAACCGTAG		
ara1	C-terminal T2A-Turboid-HA	*Tb-cytoII*	PY17X_1412750	GGAAAAAATGTATGCACAAC	TGCTCATATTCATCCACGATA	TGTAGGTAGCTATTTAGTT	ATCCATTTGAAAACCGTAG		
ara1	C-terminal 6HA	*ara1::6HA*	PY17X_1412750	GGAAAAAATGTATGCACAAC	TGCTCATATTCATCCACGATA	TGTAGGTAGCTATTTAGTT	ATCCATTTGAAAACCGTAG		
aprp2	C-terminal 6HA	*aprp2::6HA*	PY17X_1322400	GCATATATTCCGAGGGAAAAT	TACGACGATATATCGCCAAACT	TTATTAAACTATTTTTCACGAT	GCTATTTTCGTTTAATGTGTC		
aprp4	C-terminal 6HA	*aprp4::6HA*	PY17X_1214800	GTTAACACAACGTCATAAGT	ACGTTTTCTTGGAATATATATT	ATGGATAATTTATTATATTACAT	AGTTTGGCGATATATCGTCGTATAT		
**Primers for transient episomal expression**									
**Gene name**	**Gene ID**	**Description**	**CDS**				**Product length (bp)**		
			**Forward primer**		**Reverse primer**				
apr2-N	PY17X_1339500	1-600 aa	AACGACAAAGCTAGCATGAGAAAAAAAAGTGAAATTTCCAAA		AGAACCGGAGCTAGCTTCATTTGGGAATTTATCTATTTTACAT		1800		
apr2-M	PY17X_1339500	501-1100 aa	AACGACAAAGCTAGCATGGTCGAAAATATATCAAATGAAT		AGAACCGGAGCTAGCAATCAAATTTGAGGTTTTAT		1800		
apr2-C	PY17X_1339500	1001-1384 aa	AACGACAAAGCTAGCATGGTGTCACAAGGATTTACTCATGCAA		AGAACCGGAGCTAGCGTTTAAATGACGTGATGCATATGAAT		1182		
ara1	PY17X_1412750	Full lenth of coding sequence	AACGACAAAGCTAGCATGTCTATCGTAATTGCCCCAAGT		AGAACCGGAGCTAGCTATTATTATGGTTTTTCCTCCTATT		189		
aprp1	PY17X_0721000		AACGACAAAGCTAGCATGAATTTACTTCATGCATATAT		AGAACCGGAGCTAGCATTGAATCTGCAAAAAAAAAAAAAAATTT		1769		
aprp2	PY17X_1322400		AACGACAAAGCTAGCATGCAACCTTTCAATTTCGTAACACA		AGAACCGGAGCTAGCCATTTGAATATTTTTATTCAAGT		456		
aprp3	PY17X_0402200		AACGACAAAGCTAGCATGAGAGTCAGTATTTTAAGATTT		AGAACCGGAGCTAGCTTTTGATGTTTTTGATTTTTGCTTTAT		946		
aprp4	PY17X_1322300		AACGACAAAGCTAGCATGGATAATTTATTATATTACATAAT		AGAACCGGAGCTAGCCCCTGTATGATTATTTGAATATT		525		
aprp5	PY17X_0807500		AACGACAAAGCTAGCATGGCTTACCATTTATTCAAGAAAT		AGAACCGGAGCTAGCTTTATGTTCGTTTTCATCTGGGTTT		5634		
aprp6	PY17X_1336300		AACGACAAAGCTAGCATGAACACAATAGAATTAAGTTAT		AGAACCGGAGCTAGCTTCCAAATTAAATTTATATTTATGCAT		1097		
aprp7	PY17X_1111000		AACGACAAAGCTAGCATGCCAAAATTTATGAGTGAAT		AGAACCGGAGCTAGCTCTTTTGAAAATAATAGGTGCGTGAAAT		963		
aprp8	PY17X_0612100		AACGACAAAGCTAGCATGTTTTTTTTTTAATAGTGAAAAACCC		AGAACCGGAGCTAGCTGAGGTCACTTGAATAACTTTTTTCT		699		
aprp9	PY17X_1322800		AACGACAAAGCTAGCATGGGTATCGATATAAATACGA		AGAACCGGAGCTAGCTCGATAAGTGCTATAGGCTCTT		2400		
apr2	PY17X_1339500	APR2-N (1-600 aa)	AACGACAAAGCTAGCATGAGAAAAAAAAGTGAAATTTCCAAA		AGAACCGGAGCTAGCTTCATTTGGGAATTTATCTATTTTACAT		1800		
apr2	PY17X_1339500	APR2-N1 (1-500 aa)	AACGACAAAGCTAGCATGAGAAAAAAAAGTGAAATTTCCAAA		AGAACCGGAGCTAGCATTATCTAAATTGTTTTTTATGGAAT		1500		
apr2	PY17X_1339500	APR2-N2 (1-400 aa)	AACGACAAAGCTAGCATGAGAAAAAAAAGTGAAATTTCCAAA		AGAACCGGAGCTAGCTATATTATGCGAATTTACAACATTATCCT		1200		
apr2	PY17X_1339500	APR2-N3 (1-300 aa)	AACGACAAAGCTAGCATGAGAAAAAAAAGTGAAATTTCCAAA		AGAACCGGAGCTAGCGGAAGAGTCAGTTTTATTTTCT		900		
apr2	PY17X_1339500	APR2-N4 (1-200 aa)	AACGACAAAGCTAGCATGAGAAAAAAAAGTGAAATTTCCAAA		AGAACCGGAGCTAGCATGGACTTCAGGTTTTTCAGA		600		
apr2	PY17X_1339500	APR2-N5 (1-100 aa)	AACGACAAAGCTAGCATGAGAAAAAAAAGTGAAATTTCCAAA		AGAACCGGAGCTAGCTGTTTTATTTTCTTCAAAATTTTTAT		300		
apr2	PY17X_1339500	APR2-N6 (1-50 aa)	AACGACAAAGCTAGCATGAGAAAAAAAAGTGAAATTTCCAAA		AGAACCGGAGCTAGCATTAGCCCCTGAATATACTTCTAT		150		
apr2	PY17X_1339500	APR2-N7 (50-200 aa)	AACGACAAAGCTAGCAATAAAGCCTACTATGAAAATTATAT		AGAACCGGAGCTAGCATGGACTTCAGGTTTTTCAGA		453		

Supplementary Table 2. List of genetically modified parasite strains used in this study

**Strain**	**Description**			**Resource**
17XNL	non-lethal strain *Plasmodium yoelii* 17XNL			NIH
**Parasite with gene deletion**	**Gene ID**	**Parental strain**	**Description**	**Resource**
Δapr2	PY17X_1339500	17XNL	Deleted the whole coding sequence of *apr2* in the 17XNL parasite	This study
Δapr2n	PY17X_1339500	17XNL	Deleted the N-terminal 601bp (1-601) of *apr2* and caused frame- shift mutation in the 17XNL parasite	This study
APR2-ΔN	PY17X_1339500	*apr2::GFP*	Deleted the N-terminal 599 aa (2-600) of *apr2*	This study
Δapr2;Tb-ARA1	PY17X_1339500	*Tb-ARA1*	Deleted the whole coding sequence of *apr2* in the *Tb-ARA1* parasite	This study
Δara1	PY17X_1412750	17XNL	Deleted the whole coding sequence of *ara1* in the 17XNL parasite	This study
Δaprp2	PY17X_1322400	17XNL	Deleted the whole coding sequence of *aprp2* in the 17XNL parasite	This study
Δaprp4	PY17X_1214800	17XNL	Deleted the whole coding sequence of *aprp4* in the 17XNL parasite	This study
**Gene compelementation strains**	**Gene ID**	**Parental strain**	**Description**	**Resource**
comp	PY17X_1339500	*Δapr2n*	Complementation of HA tagged *apr2* sequence (1-601 bp) in the *Δapr2n* parasite	This study
**Parasites with gene tagging**	**Gene ID**	**Parental strain**	**Description**	**Resource**
apr2::6HA	PY17X_1339500	17XNL	C-terminally tagged with 6HA in the 17XNL parasite	This study
6HA::apr2	PY17X_1339500	17XNL	N-terminally tagged with 6HA in the 17XNL parasite	This study
apr2::GFP	PY17X_1339500	17XNL	C-terminally tagged with GFP in the 17XNL parasite	This study
GFP::apr2	PY17X_1339500	17XNL	N-terminally tagged with GFP in the 17XNL parasite	This study
sas6l::4Myc	PY17X_1416600	*apr2::6HA*	C-terminally tagged with 4Myc in the *apr2::6HA* parasite	This study
myosinB::4Myc	PY17X_1104400	*apr2::6HA*	C-terminally tagged with 4Myc in the *apr2::6HA* parasite	This study
APR2-T2A	PY17X_1339500	17XNL	T2A insertion between aa 845 and 846	This study
Tb-APR2	PY17X_1339500	17XNL	C-terminally tagged with TurboID in the 17XNL parasite	This study
Tb-CytoI	PY17X_1339500	17XNL	C-terminally tagged with T2A-TurboID in the 17XNL parasite	This study
Tb-ARA1	PY17X_1412750	17XNL	C-terminally tagged with TurboID in the 17XNL parasite	This study
Tb-CytoII	PY17X_1412750	17XNL	C-terminally tagged with T2A-TurboID in the 17XNL parasite	This study
ara1::6HA	PY17X_1412750	*apr2::GFP*	C-terminally tagged with 6HA in the *apr2::GFP* parasite	This study
aprp2::6HA	PY17X_1322400	*apr2::GFP*	C-terminally tagged with 6HA in the *apr2::GFP* parasite	This study
aprp4::6HA	PY17X_1214800	*apr2::GFP*	C-terminally tagged with 6HA in the *apr2::GFP* parasite	This study
**Gene transient expression in the parasite**	**Parental strain**		**Description**	**Resource**
APR2-N::6HA	17XNL		N-terminal part (1-600 aa) of the APR2 tagged with 6HA in the 17XNL parasite	This study
APR2-M::6HA	17XNL		Middle part (501-1,100 aa) of the APR2 tagged with 6HA in the 17XNL parasite	This study
APR2-C::6HA	17XNL		C-terminal part (1,001-1,394 aa) of the APR2 tagged with 6HA in the 17XNL parasite	This study
APR2-N::6HA	*Δapr2*		N-terminal part (1-600 aa) of the APR2 tagged with 6HA in the *Δapr2* parasite	This study
APR2-M::6HA	*Δapr2*		Middle part (501-1,100 aa) of the APR2 tagged with 6HA in the *Δapr2* parasite	This study
APR2-C::6HA	*Δapr2*		C-terminal part (1,001-1,394 aa) of the APR2 tagged with 6HA in the *Δapr2* parasite	This study
APR2-N(1-500 aa)::GFP	17XNL		N-terminal part (1-500 aa) of the APR2 tagged with GFP in the 17XNL parasite	This study
APR2-N(1-400 aa)::GFP	17XNL		N-terminal part (1-400 aa) of the APR2 tagged with GFP in the 17XNL parasite	This study
APR2-N(1-300 aa)::GFP	17XNL		N-terminal part (1-300 aa) of the APR2 tagged with GFP in the 17XNL parasite	This study
APR2-N(1-200 aa)::GFP	17XNL		N-terminal part (1-200 aa) of the APR2 tagged with GFP in the 17XNL parasite	This study
APR2-N(1-100 aa)::GFP	17XNL		N-terminal part (1-100 aa) of the APR2 tagged with GFP in the 17XNL parasite	This study
APR2-N(50-200 aa)::GFP	17XNL		N-terminal part (50-200 aa) of the APR2 tagged with GFP in the 17XNL parasite	This study
APRp1::6HA	*apr2::GFP*		Full length of the APRp1 tagged with 6HA in the *apr2::gfp* parasite	This study
APRp2::6HA	*apr2::GFP*		Full length of the APRp2 tagged with 6HA in the *apr2::gfp* parasite	This study
APRp3::6HA	*apr2::GFP*		Full length of the APRp3 tagged with 6HA in the *apr2::gfp* parasite	This study
APRp4::6HA	*apr2::GFP*		Full length of the APRp4 tagged with 6HA in the *apr2::gfp* parasite	This study
APRp5::6HA	*apr2::GFP*		Full length of the APRp5 tagged with 6HA in the *apr2::gfp* parasite	This study
APRp6::6HA	*apr2::GFP*		Full length of the APRp6 tagged with 6HA in the *apr2::gfp* parasite	This study
APRp7::6HA	*apr2::GFP*		Full length of the APRp7 tagged with 6HA in the *apr2::gfp* parasite	This study
APRp8::6HA	*apr2::GFP*		Full length of the APRp8 tagged with 6HA in the *apr2::gfp* parasite	This study
APRp9::6HA	*apr2::GFP*		Full length of the APRp9 tagged with 6HA in the *apr2::gfp* parasite	This study
