# Supplementary material for: Apical anchorage and stabilization of subpellicular microtubules by apical polar ring ensures Plasmodium ookinete infection in mosquito
Source: Nat Commun. 2022 Dec 3;13:7465. doi: 10.1038/s41467-022-35270-w (PMC9719560; doi:10.1038/s41467-022-35270-w)
Supplement: Supplementary file 3 — Description of Additional Supplementary Files [file 41467_2022_35270_MOESM3_ESM.pdf]

Description of supplementary datasets

Apical anchorage and stabilization of subpellicular microtubules by apical polar ring ensures *Plasmodium* ookinete infection in mosquito

Pengge Qian[1], Xu Wang[1], Cuirong Guan[2], Xin Fang[1], Mengya Cai[1], Chuan-qi Zhong[1], Yong Cui[1], Yanbin Li[1], Luming Yao[1], Huiting Cui[1,*], Kai Jiang[2,*], Jing Yuan[1,*]

1. Supplementary Data 1 List of Tb-APR2 interacting protein candidates identified in this study

2. Supplementary Data 2 List of Tb-ARA1 interacting protein candidates identified in this study
