# Supplementary material for: Apical anchorage and stabilization of subpellicular microtubules by apical polar ring ensures Plasmodium ookinete infection in mosquito
Source: Nat Commun. 2022 Dec 3;13:7465. doi: 10.1038/s41467-022-35270-w (PMC9719560; doi:10.1038/s41467-022-35270-w)
Supplement: Supplementary file 6 — Reporting Summary [file 41467_2022_35270_MOESM6_ESM.pdf]

nature portfolio

Corresponding author(s): Jing Yuan

Last updated by author(s): Nov 17, 2022

Reporting Summary

Nature Portfolio wishes to improve the reproducibility of the work that we publish. This form provides structure for consistency and transparency in reporting. For further information on Nature Portfolio policies, see our Editorial Policies and the Editorial Policy Checklist.

Statistics

For all statistical analyses, confirm that the following items are present in the figure legend, table legend, main text, or Methods section.

n/a	Confirmed	
☐	☒	The exact sample size (n) for each experimental group/condition, given as a discrete number and unit of measurement
☐	☒	A statement on whether measurements were taken from distinct samples or whether the same sample was measured repeatedly
☐	☒	The statistical test(s) used AND whether they are one- or two-sided<br>*Only common tests should be described solely by name; describe more complex techniques in the Methods section.*
☒	☐	A description of all covariates tested
☒	☐	A description of any assumptions or corrections, such as tests of normality and adjustment for multiple comparisons
☐	☒	A full description of the statistical parameters including central tendency (e.g. means) or other basic estimates (e.g. regression coefficient) AND variation (e.g. standard deviation) or associated estimates of uncertainty (e.g. confidence intervals)
☐	☒	For null hypothesis testing, the test statistic (e.g. F, t, r) with confidence intervals, effect sizes, degrees of freedom and P value noted<br>*Give P values as exact values whenever suitable.*
☒	☐	For Bayesian analysis, information on the choice of priors and Markov chain Monte Carlo settings
☒	☐	For hierarchical and complex designs, identification of the appropriate level for tests and full reporting of outcomes
☒	☐	Estimates of effect sizes (e.g. Cohen's d, Pearson's r), indicating how they were calculated

Our web collection on statistics for biologists contains articles on many of the points above.

Software and code

Policy information about availability of computer code

Data collection	Fluorescent images were acquired using Zeiss LSM 880 and Zeiss LSM 980 confocal microscopy; DNA electrophoresis were imaged with Gel Image System (Tanon-2500); Electron microscopy was performed with using the Hitachi HT-7800 electron microscope.
Data analysis	Fluorescent images were processed in ZEN blue (Lite2.3) ; Imaris (Imaris X64 9.2.0) and Image J (Fiji, 1.52v) were used for image quantification analysis; Statistical analysis was performed by GraphPad Prism (version 8.0).

For manuscripts utilizing custom algorithms or software that are central to the research but not yet described in published literature, software must be made available to editors and reviewers. We strongly encourage code deposition in a community repository (e.g. GitHub). See the Nature Portfolio guidelines for submitting code & software for further information.

Data

Policy information about availability of data

All manuscripts must include a data availability statement. This statement should provide the following information, where applicable:

- Accession codes, unique identifiers, or web links for publicly available datasets
- A description of any restrictions on data availability
- For clinical datasets or third party data, please ensure that the statement adheres to our policy

Mass spectrometry proteomics data have been deposited to the ProteomeXchange Consortium (http://proteomecentral.proteomexchange.org) via the iProX partner repository with the dataset identifier PXD038209. All other relevant data in this study are submitted as supplementary source files. Source data are provided with this paper.

March 2021

Field-specific reporting

Please select the one below that is the best fit for your research. If you are not sure, read the appropriate sections before making your selection.

☒ Life sciences ☐ Behavioural & social sciences ☐ Ecological, evolutionary & environmental sciences

For a reference copy of the document with all sections, see nature.com/documents/nr-reporting-summary-flat.pdf

Life sciences study design

All studies must disclose on these points even when the disclosure is negative.

Sample size	Sample size was determined according to the similar kind of experiments previously published in the lab, including "An intracellular membrane protein GEP1 regulates xanthurenic acid induced gametogenesis of malaria parasites", Nature Communications, 2020; "A malaria parasite phospholipid flippase safeguards midgut traversal of ookinetes for mosquito transmission", Science Advances, 2021; "ISP1-Anchored Polarization of GCβ/CDC50A Complex Initiates Malaria Ookinete Gliding Motility", Current Biology, 2018. These sample sizes were sufficient to carry out the experiments with sufficient statistics and standard using t-test and other test.
Data exclusions	No data was exclued from the analyses.
Replication	The data reported were generated using at least three different biological replicate in most required experiments. At least three technical replicate per parasite were tested for each condition evaluated. All attempts at replication were successful.
Randomization	For parasite infection assay, the mice and mosquitoes were randomly divided into corresponding groups. For other biochemical experiments, samples were also randomly allocated into experimental groups.
Blinding	The investigators were not blinded for experiments comprising objective measurements such as western blotting and IFA. However, investigator bias is not considered to contribute to the data because the investigator was blinded at the time of data analysis.<br>In mass spectrometry analysis, investigators performing mass spectrometry data collection and data analysis were blinded to experimental groups (including sample name and groups) ahead of final results were done.

Reporting for specific materials, systems and methods

We require information from authors about some types of materials, experimental systems and methods used in many studies. Here, indicate whether each material, system or method listed is relevant to your study. If you are not sure if a list item applies to your research, read the appropriate section before selecting a response.

Materials & experimental systems

n/a	Involved in the study	
☐	☒	Antibodies
☐	☒	Eukaryotic cell lines
☒	☐	Palaeontology and archaeology
☐	☒	Animals and other organisms
☒	☐	Human research participants
☒	☐	Clinical data
☒	☐	Dual use research of concern

Methods

n/a	Involved in the study	
☒	☐	ChIP-seq
☒	☐	Flow cytometry
☒	☐	MRI-based neuroimaging

Antibodies

Antibodies used	The primary antibodies used were: rabbit anti-HA (Cell Signaling Technology (CST), cat#3724S, 1:1,000 for immunoblotting (IB), 1:500 for immunofluorescence (IF), 1:500 for immunoprecipitation (IP)), mouse anti-HA(CST, cat#2367S, 1:500 for IF), rabbit anti-Myc (CST, cat#2272S, 1:1,000 for IB), mouse anti-Myc (CST, cat#2276S, 1:500 for IF), mouse anti-α-tubulin II (Sigma-Aldrich, cat#T6199, 1:1,000 for IF, 1:1,000 for IB), mouse anti-β-tubulin (Sigma-Aldrich, cat#T5201, 1:1,000 for IF, 1:1,000 for IB), rabbit anti-Polyglutamate chain (PolyE) (AdipoGen, cat#AG-25B-0030, 1:1,000 for IF), rabbit anti-GFP (Abcam, cat#ab6556, 1:2,000 for IF, 1:2,000 for IB, 1:1,000 for IP) and mouse anti-GAPDH (Servicebio, cat#GB12002, 1:1,000 for IB). The secondary antibodies used were as follows: HRP-conjugated goat anti-rabbit IgG (Abcam, cat#ab6721, 1:5,000 for IB), HRP-conjugated goat anti-mouse IgG (Abcam, cat#ab6789, 1:5,000 for IB), Alexa 555 goat anti-rabbit IgG (Thermo Fisher Scientific, cat#A21428, 1:1,000 for IF), Alexa 488 goat anti-rabbit IgG (Thermo Fisher Scientific, cat#A31566, 1:1,000 for IF), Alexa 555 goat anti-mouse IgG (Thermo Fisher Scientific, cat#A21422, 1:1,000 for IF), and Alexa 488 goat anti-mouse IgG (Thermo Fisher Scientific, cat#A11001, 1:1,000 for IF). The anti-serums, including the rabbit anti-P28 (1:1,000 for IB, 1:1,000 for IF), rabbit anti-BiP (1:1,000 for IB), rabbit anti-enolase (1:1,000 for IB), rabbit anti-GAP45 (1:1,000 for IF), rabbit anti-WARP (1:1,000 for IB), rabbit anti-CTRP (1:1,000 for IB) and rabbit anti-Chitinase (1:1,000 for IB).
Validation	All antibodies were obtained commercially. These antibodies were tested and validated by the respective company. All antibodies had validation statement provided on the website of the manufacturer.<br>rabbit anti-HA (Cell Signaling Technology (CST), cat#3724S) https://www.cellsignal.com/products/primary-antibodies/ha-tag-c29f4-

rabbit-mab/3724
mouse anti-HA (CST, cat#2367S) https://www.cellsignal.cn/products/primary-antibodies/ha-tag-6e2-mouse-mab/2367
rabbit anti-Myc (CST, cat#2272S) https://www.cellsignal.cn/products/primary-antibodies/myc-tag-antibody/2272?N=4294966899&fromPage=plp
mouse anti-Myc (CST, cat#2276S) https://www.cellsignal.cn/products/primary-antibodies/myc-tag-9b11-mouse-mab/2276
mouse anti-α-tubulin II (Sigma-Aldrich, cat#T6199) https://www.sigmaaldrich.cn/CN/zh/product/sigma/t6199
mouse anti-β-tubulin (Sigma-Aldrich, cat#T5201) https://www.sigmaaldrich.cn/CN/zh/product/sigma/t5201
rabbit anti-Polyglutamate chain (PolyE) (AdipoGen, cat#AG-25B-0030) https://adipogen.com/ag-25b-0030-anti-polyglutamate-chain-polye-pab-in105.html/
rabbit anti-GFP (Abcam, cat#ab6556) https://www.abcam.com/gfp-antibody-ab6556.html
mouse anti-GAPDH (Servicebio, cat#GB12002) https://www.servicebio.cn/goodsdetail?id=588

All antiserums used in this study have been well-determined:
BiP anti-rabbit, P28 anti-rabbit- doi: 10.1016/j.cub.2018.06.069
enolase anti-rabbit, CTRP anti-rabbit, WARP anti-rabbit, chitinase anti-rabbit, GAP45 anti-mouse -doi: 10.1126/sciadv.abf6015.

Eukaryotic cell lines

Policy information about cell lines

Cell line source(s)	The HEK-293T (human embryonic kidney cell line) and MRC-5 (Medical Research Council cell strain 5) were obtained from ATCC.
Authentication	Cell line was authenticated based on unique morphology and growth characteristics.
Mycoplasma contamination	MRC-5 and HEK-293T cells were tested negative for mycoplasma-contamination using Mycoplasma Detection Kit (InvivoGen).
Commonly misidentified lines (See ICLAC register)	No commonly misidentified cell lines were used in the study.

Animals and other organisms

Policy information about studies involving animals; ARRIVE guidelines recommended for reporting animal research

Laboratory animals	ICR mice (female, 5 to 6 weeks old) were housed in Animal Care Center of Xiamen University and kept at 22–24°C under a 12 h light/dark cycle at a constant relative humidity of 45%–65%.
Wild animals	The study did not involve wild animals.
Field-collected samples	No field-collected samples were used in this study.
Ethics oversight	All experiments were reviewed and approved by the Committee for Care and Use of Laboratory Animals of Xiamen University (XMULAC20140004).

Note that full information on the approval of the study protocol must also be provided in the manuscript.
