# Supplementary material for: Apical anchorage and stabilization of subpellicular microtubules by apical polar ring ensures Plasmodium ookinete infection in mosquito
Source: Nat Commun. 2022 Dec 3;13:7465. doi: 10.1038/s41467-022-35270-w (PMC9719560; doi:10.1038/s41467-022-35270-w)

Fig. 1G

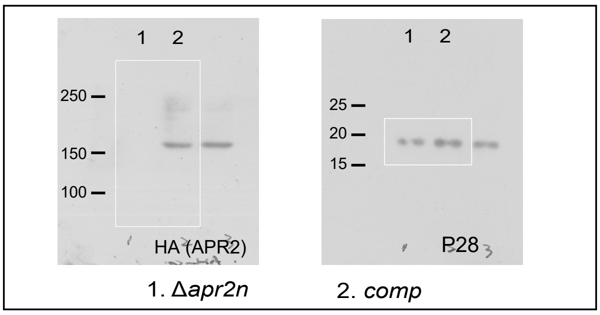

Fig. 3C

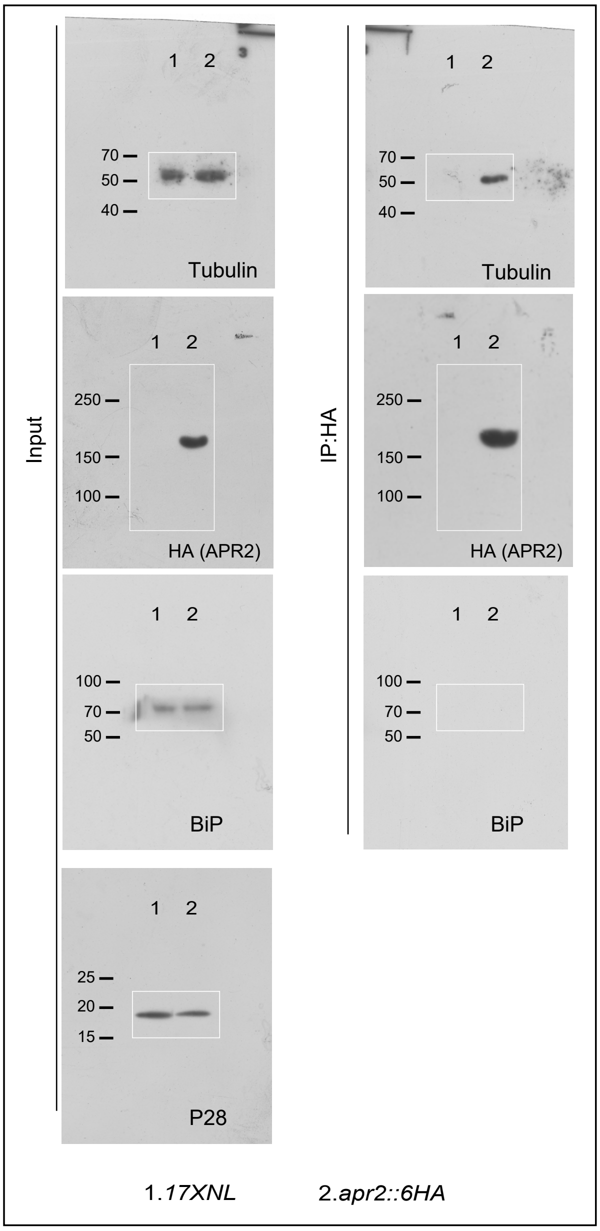

Fig. 3D

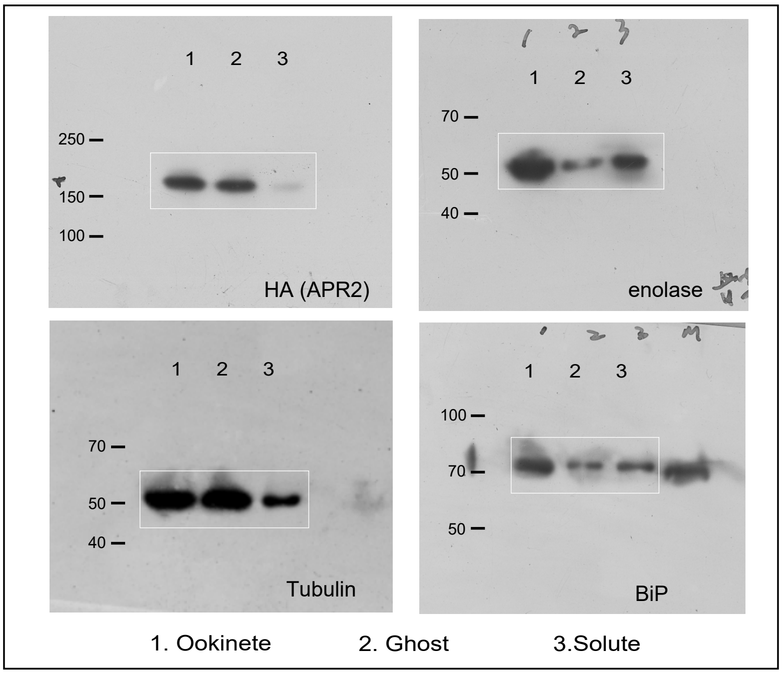

Fig. 4C

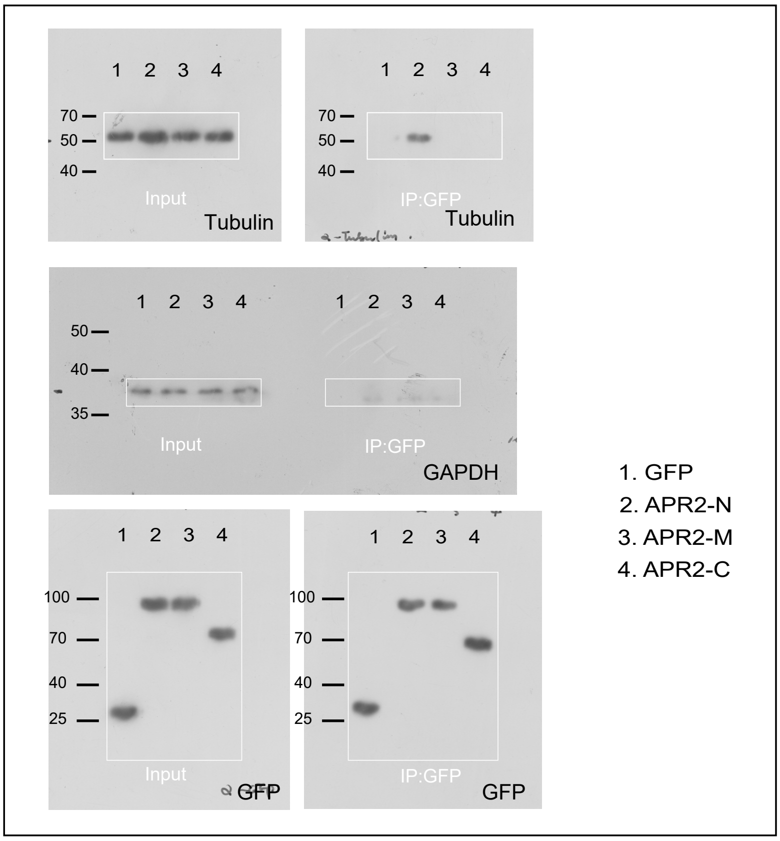

Fig. 5D

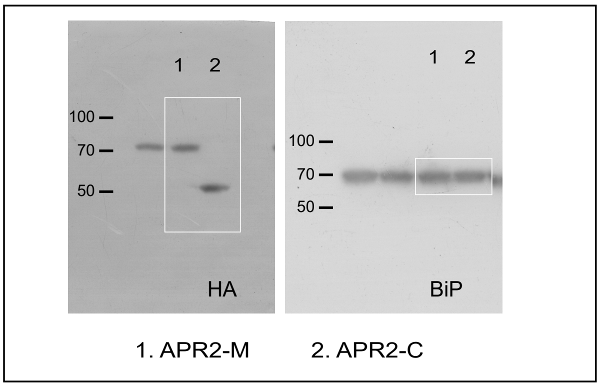

Fig. 5H

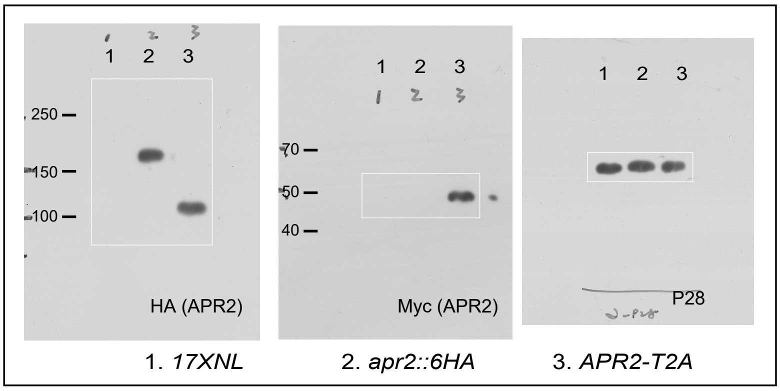

Fig. 7G

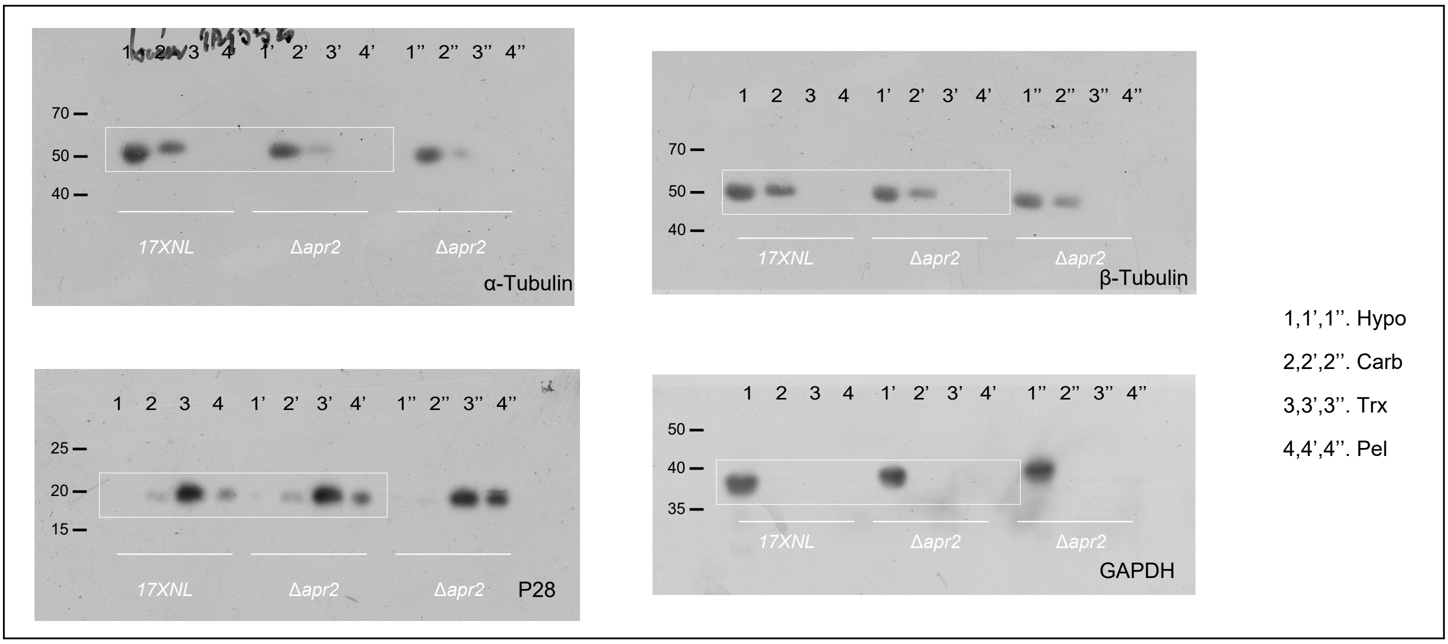

FigS. 2A

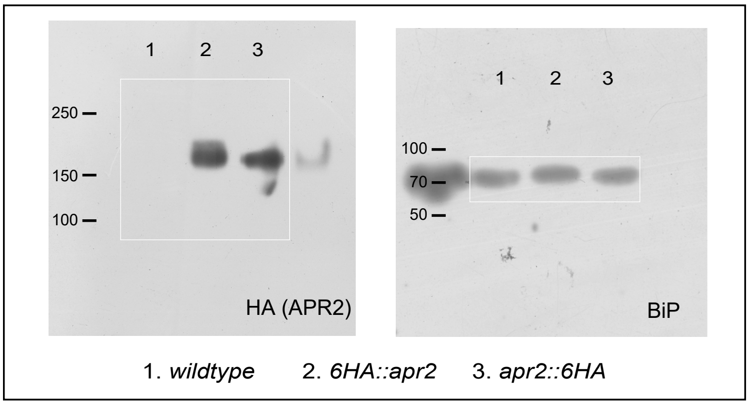

FigS. 2B

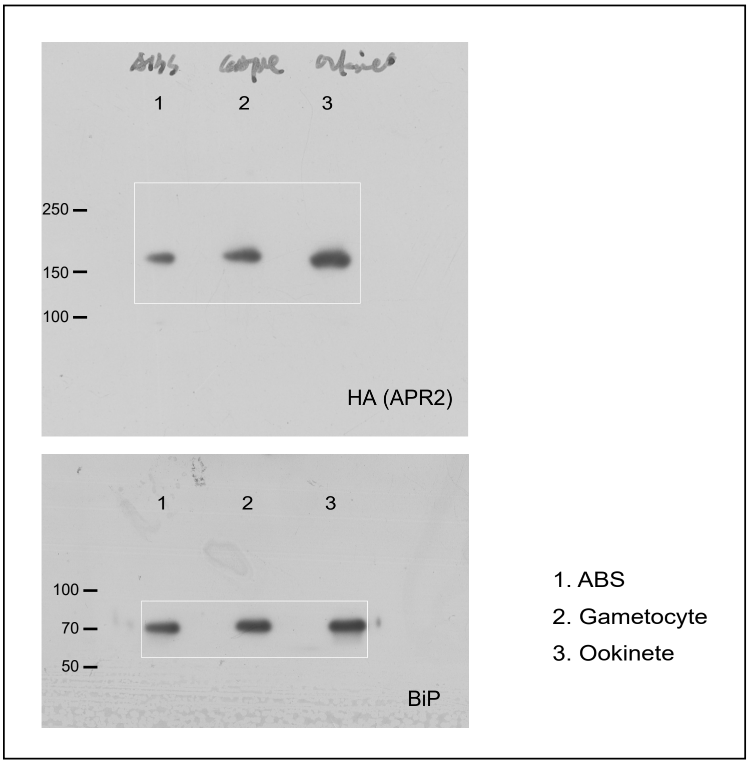

FigS. 7B

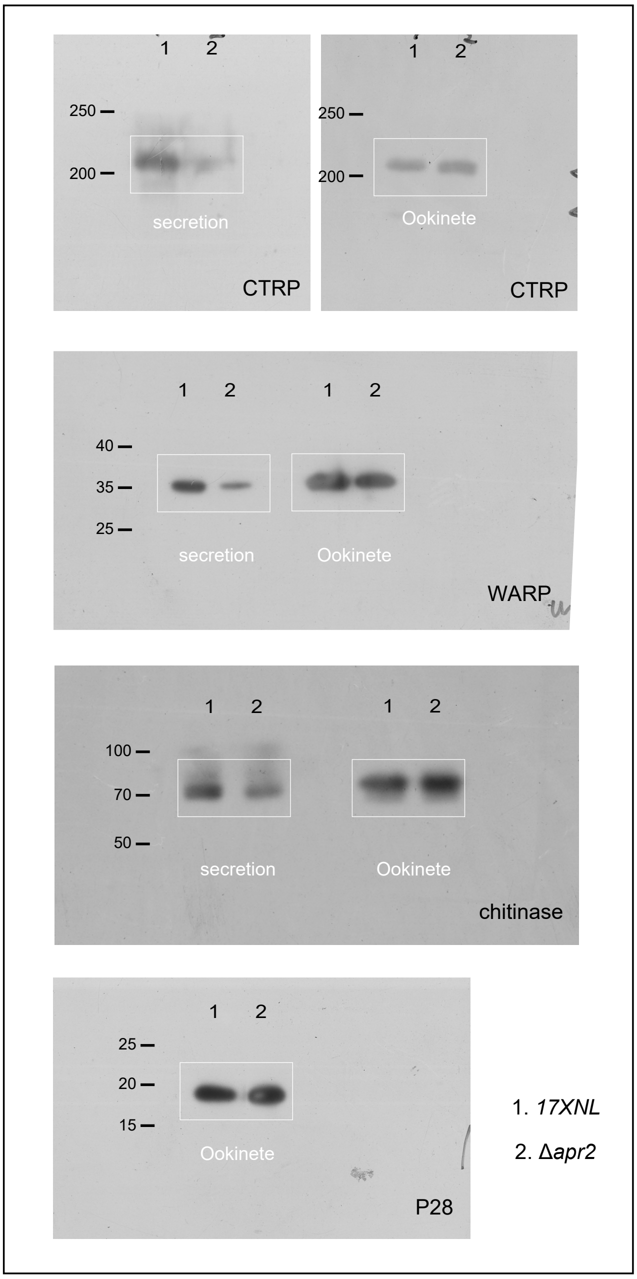

Supplement: Supplementary file 7 — Source Data [file 41467_2022_35270_MOESM7_ESM.zip › Source data/raw WB data.pdf]
